# An Unexpected Seasonal Cycle in U.S. Oil and Gas Methane Emissions

**DOI:** 10.1021/acs.est.4c14090

**Published:** 2025-05-14

**Authors:** Lei Hu, Arlyn E. Andrews, Stephen A. Montzka, Scot M. Miller, Lori Bruhwiler, Youmi Oh, Colm Sweeney, John B. Miller, Kathryn McKain, Sergio Ibarra Espinosa, Kenneth Davis, Natasha Miles, Marikate Mountain, Xin Lan, Andy Crotwell, Monica Madronich, Thomas Mefford, Sylvia Michel, Sander Houwelling

**Affiliations:** † Global Monitoring Laboratory, US National Oceanic and Atmospheric Administration, Boulder, Colorado 80305, United States; ‡ Department of Environmental Health and Engineering, Johns Hopkins University, Baltimore, Maryland 21218, United States; § Cooperative Institute for Research in Environmental Sciences, 1877 University of Colorado-Boulder, Boulder, Colorado 80309, United States; ∥ Department of Meteorology and Atmospheric Science, The Pennsylvania State University, University Park, Pennsylvania 16802, United States; ⊥ Earth and Environmental Systems Institute, The Pennsylvania State University, University Park, State College, Pennsylvania 16802, United States; # 322779 Atmospheric and Environmental Research Inc., Lexington, Massachusetts 02421, United States; ¶ Institute for Arctic and Alpine Research, University of Colorado-Boulder, Boulder, Colorado 80309, United States; ∇ Department of Earth Sciences, 1190 Vrije Universiteit Amsterdam, 1081 HV Amsterdam, the Netherlands

**Keywords:** greenhouse gas, anthropogenic emissions, inverse modeling, atmospheric observations, seasonal variation, top-down estimates

## Abstract

Accurate quantification of methane (CH_4_) emissions is essential for understanding changes in its atmospheric abundance. Atmospheric observations can supply independent emission information that complements and strengthens inventory-based estimates. In this study, we quantified annual and monthly U.S. CH_4_ emissions in 2008–2021 using inverse modeling of ground and airborne measurements at sites across the U.S. with 10–12 km atmospheric transport simulations. While the magnitude, spatial distribution, and trend of the estimated CH_4_ emissions align with some previous studies, our results reveal an unexpected seasonal cycle in CH_4_ emissions from the oil and gas sector, where wintertime emissions are about 40 (20–50, 2σ) % higher than summertime. This seasonality is supported by methane and propane measurements at these same sites, as well as methane isotope measurements made from an independent aircraft campaign over the U.S. Although the exact cause of this emission seasonality is unclear, its spatial distribution indicates that the enhanced CH_4_ emissions are primarily from natural gas production regions, and to a lesser extent, from natural gas consumption in winter.

## Graphical abstract

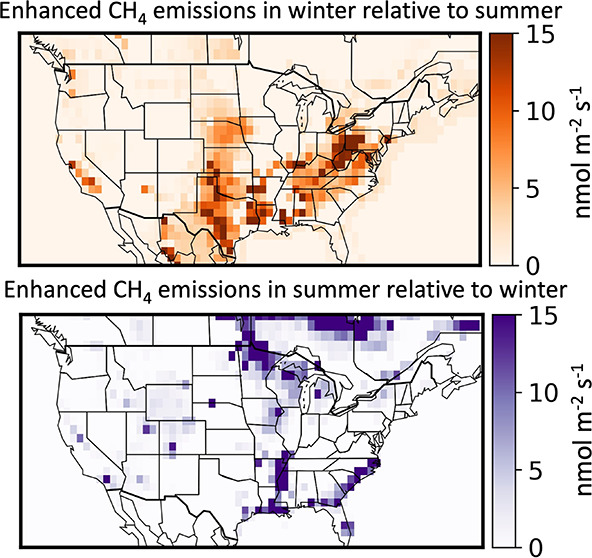

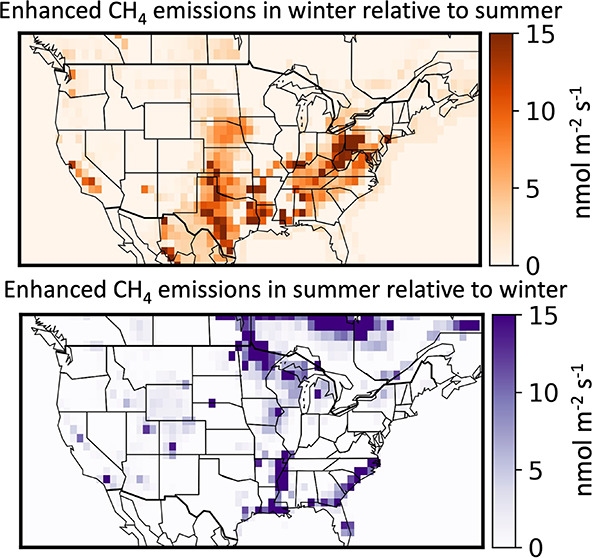

## 1 Introduction

Methane (CH_4_) is a potent greenhouse gas and currently contributes the second largest radiative forcing after CO_2_ ^1^ when compared to other long-lived greenhouse gases. It has a global warming potential (GWP) of 28 over a 100 year time horizon.^2^ The atmospheric abundance or mole fraction of CH_4_ has nearly tripled since the preindustrial era.^3^ Currently, the global atmospheric CH_4_ continues to rise and has been increasing more rapidly since 2014 ^4^ due to increases in its global emissions^5^ and reduction in its chemical sink.^6,7^ Because of the rapid growth of CH_4_ abundance in the atmosphere and its potency in influencing climate, CH_4_ has contributed to one-third of gross warming induced by all long-lived greenhouse gases (GHGs) since the preindustrial era.^8^ CH_4_ only has a lifetime of 9.1–11.8 years in the atmosphere, which is much shorter than other major GHGs such as CO_2_, N_2_O, and many fluorinated GHGs.^1^ Therefore, reducing CH_4_ emissions can result in a near-term reduction in its atmospheric abundance and generate prompt climate benefits.^9^ Because of this, the global methane pledge, endorsed by about 150 countries so far, aims to reduce global anthropogenic CH_4_ emissions by at least 30% below 2020 levels by 2030.^10^

CH_4_ is not only a powerful greenhouse gas, but also the primary component in natural gas. Identification of CH_4_ emission processes can inform the development of mitigation strategies for reducing emissions and, in some instances, economic losses. Furthermore, accurate quantification of emission magnitudes helps evaluate the effectiveness of mitigation efforts. In the U.S., the Environmental Protection Agency (EPA)’s Greenhouse Gas Inventory (GHGI)^11^ is the official emission tracking system for annual anthropogenic emissions of CH_4_ and other GHGs. It contains estimates from identified emission processes and is reported to the United Nations Framework Convention on Climate Change each year. As highlighted in the 2019 Refinement to the 2006 IPCC Guidelines for National Greenhouse Gas Inventories,^12^ estimates provided by atmospheric measurements are key to independently evaluate the accuracy of the EPA’s GHGI and provide emission estimates at higher spatial and temporal resolutions. So far, there have been many atmosphere-based inverse modeling studies that use atmospheric CH_4_ measurements to estimate national-scale CH_4_ emissions,^13–17^ including for the U.S.^13,18,19^ Studies also have used smaller-scale campaigns to provide independent evaluation of the EPA’s GHGI from large oil and gas basins down to city and device scales.^20^ Consistently, all of these independent atmospheric studies suggest a substantial underestimation of EPA’s GHGI for oil and gas CH_4_ emissions, ranging from a factor of 1.5 on a national scale to a factor of 1000 on individual devices.^20,21^ Besides independently evaluating the GHGI reported emission magnitudes, the atmospheric observation-based estimates are also demonstrated to be useful in providing additional insights on possible emission sources beyond what have been identified in the GHGI.^18,22,23^ In this study, we used atmospheric CH_4_ observations made across the U.S. for 2008–2021 with a high-resolution regional Lagrangian inverse model to estimate U.S. national and regional CH_4_ emissions on annual and monthly scales.

## 2 Methods

### 2.1 Inverse Modeling Overview

This study uses CH_4_ measurements made by the U.S. National Oceanic and Atmospheric Administration (NOAA) Global Monitoring Laboratory (GML) from whole-air flask samples collected at 0–6 km above the ground level from Earth’s surface, tall towers,^24^ and aircraft^25^ primarily located in the U.S. between 2008 and 2021 (Figure S1). Air samples were collected every other day from towers, and about every two to 4 weeks at aircraft profiling sites. At a couple of sites such as in Utah and Florida, only weekly flask samples get collected.

To estimate U.S. CH_4_ emissions, we modified the regional inverse modeling framework that we developed for other gases.^26–29^ For a long-lived trace gas that has zero or negligible chemical loss, the measured mole fractions (χ_obs_ in parts per billion or ppb) can be estimated as the sum of its background mole fractions upwind of the measurement locations (χ_bg_ in ppb) plus any enhancements (or depletions) caused by surface emissions (or uptake) (*s* in nmol m^–2^ s^–1^) (eq 1).

1 $$\chi_{obs}=\chi_{bg}+Hs+\varepsilon$$

where *H* represents the sensitivity of each atmospheric measurement to surface emissions (or uptake) (also called “footprints”)^30^ and has a unit of ppb (nmol m^–2^ s^–1^)^−1^. It was calculated by the HYbrid Single-Particle Lagrangian Integrated Trajectory model^31^ driven by the North American Mesoscale Forecast System nested with the Global Forest System (HYSPLIT-NAMS/GFS) and the Stochastic Time-Inverted Lagrangian Transport model driven by the Weather Research and Forecasting (WRF-STILT) model (Supporting Information Text S1). ε denotes random Gaussian errors of the estimated mole fractions relative to observations. It includes random errors in the background estimates, footprints, and the estimated surface emissions.

A Bayesian inverse modeling approach is deployed to optimize scaling factors of a priori emissions at 1° × 1° × 1 week resolution (eq 2).

2 $$\lambda =\lambda_{p}+QK^{T}{\left(KQK^{T}+R\right)}^{-1}\left(z-K\lambda_{p}\right)$$

where λ (unitless) represents the state vector of optimized or posterior scaling factors. It has a dimension of *m*, which represents the number of grid cells to be optimized multiplying the number of time steps (see Supporting Information Text S2 and Figure S2). λ_p_ (unitless) denotes the vector of prior scaling factors, which consists of *m* number of ones. *z* represents mole fraction enhancements related to surface emissions from grid cells that will be optimized (unit: ppb). It is calculated from χ_obs_ – χ_bg_ – *Hs*_unopt_, where *s*_unopt_ represents areas adjacent to the region of interest but not optimized in inversions (e.g., the lower part of Mexico, Figure S2) (unit: nmol m^–2^ s^–1^). *K* denotes the Jacobian matrix or the sensitivity matrix of *z* to λ, which is calculated by *Hs*_p_ and has a unit in ppb; *s*_p_ represents the initial guess or a priori emissions over grid cells that will be optimized in inversions (Figure S2) (unit: nmol m^–2^ s^–1^). *R* (unit: ppb^2^) and *Q* (unitless) denote the model-data mismatch error covariance matrix and the error covariance matrix of prior scaling factors, which were estimated by maximum likelihood estimation^32^ (see Supporting Information Text S3).

Unlike many gases that we previously applied the inverse modeling framework to (such as many fluorinated gases or CO_2_),^26–29,33^ CH_4_ has a relatively shorter atmospheric lifetime.^1^ About 95% of its loss in the troposphere is due to its chemical reaction with hydroxyl radicals (OH) and 5% is related to reaction with tropospheric chlorine.^5^ In the past, regional Lagrangian inverse modeling studies often neglected the chemical loss of CH_4_ ^18,23^ due to the short residence time of air particles within the model domain. In this study, we accounted for the chemical loss of CH_4_ to OH and quantified its impact on national CH_4_ emission estimates based on the method described below.

The loss of CH_4_ to OH is a second-order chemical reaction. When not considering emissions, the loss rate of CH_4_ in the troposphere can be estimated by

3 $$\frac{d\chi_{CH_{4}}}{dt}=-\kappa \chi_{CH_{4}}c_{OH}$$

where, $\chi_{CH_{4}}$ represents atmospheric CH_4_ mole fraction (ppb); *c*_OH_ denotes atmospheric concentration of OH in molecules cm^–3^; κ denotes the reaction rate constant (unit: (molecules cm^–3^)^−1^ s^–1^); *t* represents the duration CH_4_ has reacted with OH (unit: s). Given eq 3 and an initial atmospheric CH_4_ mole fraction of $\chi_{CH_{4},t_{0}}$ , we can estimate the CH_4_ mole fraction at time *t* ( $\chi_{CH_{4},t}$ )­

4 $$\chi_{CH_{4},t}=\chi_{CH_{4},t_{0}}\;\exp\left(\int_{t_{0}}^{t}\left(-\kappa c_{OH}\;dt\right)\right)$$

Eq 4 represents the effect of OH on the background mole fractions of CH_4_ in air from an upwind background location to a measurement site. In addition, CH_4_ reaction with OH also affects the observed enhancement associated with new emissions (*Hs*), as this loss would slightly reduce the observed enhancement, depending on the time interval between the occurrence of emissions and the time when the enhancements get measured. To include the effect of chemical losses of CH_4_ to reaction with OH in both background and new emissions, we modified eq 1

5 $$\chi_{obs}=\chi_{bg}\;\exp\left(\int_{0}^{t1}\left(-\kappa \chi_{OH}\;dt\right)\right)+H\;\exp\left(\int_{0}^{t2}\left(-\kappa \chi_{OH}\;dt\right)\right)s+\varepsilon$$

where, *t*_1_ denotes the time when air travels from a background location to a measurement site; *t*_2_ denotes the time when air travels from an emission location to a measurement site. The solution for inversion can then be modified to

6 $$\lambda =\lambda_{p}+Q{K_{OH‐corr}}^{T}{\left(K_{OH‐corr}Q{K_{OH‐corr}}^{T}+R\right)}^{-1}\left(z_{OH‐corr}-K_{OH‐corr}\lambda_{p}\right)$$

where *K*_OH‑corr_ = *H* exp­(∫_0_^*t*_2_^(−κχ_OH_ dt))*s*_p_ and *z*_OH‑corr_ = χ_obs_ – χ_bg_ exp­(∫_0_^*t*_1_^(−κχ_OH_ dt)) – *H* exp­(∫_0_^*t*_2_^(−κχ_OH_ dt))*s*_unopt_.

We evaluated the impact of OH losses on the estimated CH_4_ background and footprint (Supporting Information Text S4) using climatological monthly OH from Spivakovsky et al.^34^ that was then optimized against global atmospheric methyl chloroform observations.^35^ Our results show that OH effects on the background significantly influenced derived CH_4_ emissions, whereas OH effects on footprint had a negligible impact on emission estimation. More specifically, when OH loss was accounted for, estimated emissions were 9.8 (±1.6) Tg yr^–1^ higher in summer and only 1.7 (±2.6) Tg yr^–1^ higher in winter (Dec–Feb) compared to scenarios without OH loss (Figure S3). Consequently, neglecting OH losses could lead to an overestimation of the seasonal cycle amplitude of US CH_4_ emissions by ∼7 Tg yr^–1^ (or 14%).

### 2.2 Prior CH_4_ Emissions (*s*_p_ and *s*_unopt_)

Two prior emissions were used for inverse modeling. The first a priori was the climatological monthly posterior emissions for 2002–2006 derived from CarbonTracker-CH_4_ published in 2014 ^13^ (“CT-CH4-2014”). CT-CH4-2014 is a global Eulerian inverse model driven by the TM5 atmospheric transport model. Posterior CH_4_ emissions from CT-CH4-2014 were obtained by scaling prior emission processes (e.g., fossil fuel emissions, agriculture and waste emissions, fire emissions, oceanic emissions, and natural emissions) on large continental scales based on ground- and airborne CH_4_ observations. The second a priori (“GHGI2020 + nat”) was constructed based on multiple process-based emission products. The anthropogenic emissions in GHGI2020 + nat is from Maasakkers et al.,^36^ which is consistent with EPA’s GHGI reporting in 2020. This anthropogenic product is only available for 2012–2018. Therefore, we used the estimate in 2012 for prior emissions in 2008–2011 and the 2018 estimate for prior emissions in 2019–2021. Wetland CH_4_ emissions were calculated at a monthly basis using the Kaplan model^37–39^ and driven by the modern-era retrospective analysis for research and applications version 2 (MERRA-2) meteorology. Monthly biomass burning emissions of CH_4_ were from the global fire emissions database version 4 (GFED4). Monthly CH_4_ fluxes from geological seeps, wild animals, termites, and soils were from the prior emissions used in the CarbonTracker-CH_4_ version 2023 (CT-CH4-2023).^17^ Monthly oceanic CH_4_ fluxes were from the posterior estimates from CT-CH4-2014: estimates before 2011 were based on CT-CH4-2014, whereas estimates from 2011 onward were taken from the CT-CH4-2014 monthly posterior estimates for 2010. The two priors, CT-CH4-2014 and GHGI2020 + nat, have different emission magnitudes, seasonality, and spatial distributions (Figure S4).

### 2.3 Estimation of Background Mole Fractions (χ_bg_)

Six sets of background mole fractions were estimated with three slightly different approaches.^26,29^ In Approach 1 (“bg”): two sets of background mole fractions were estimated from a 4-dimensional (4D) empirical background field and back-trajectories of air particles computed from HYSPLIT-NAMS/GFS or WRF-STILT models. The 4D empirical background was constructed from atmospheric measurements made in the marine boundary layer over the Pacific or Atlantic Ocean basin and free troposphere measurements made from aircraft above the North American continent (Figure S5) (see Supporting Information Text S5). With this 4D background field, we used the 500 back-trajectories computed from HYSPLIT-NAMS/GFS or WRF-STILT to determine the locations where each particle first entered the North American continent horizontally or vertically (at 5 km above sea level or asl) (Figure S2). We then averaged the background mole fractions extracted from the 4D background field at the 500 locations where air particles exited the model domain for each observation (Figure S2). In Approach 2 (“bg_corr”), we applied corrections to the background (“bg”) estimated from Approach 1. In this approach, we selected a subset of observations that had low sensitivity to surface emissions from North America and used their median difference with the estimated background as the correction for each region in each year at different vertical level (0–3 km, 3–6 km, and 6–8 km). Regions here are defined as 30–50°N, 108–130°W (region 1), 40–55°N, 86–108°W (region 2), 25–40°N, 86–108°W (region 3), 25–50°N, 64–86°W (region 4), and 54–72°N, 170–140°W (region 5). For observations outside of these regions, we applied a median difference based on all the selected background observations over North America. The 3rd approach (“bg_cams”) slightly differs from Approach 1 in terms that the posterior 4D mole fraction field from the Copernicus Atmosphere Monitoring Service (CAMS) global Eulerian inversion system (version v21r1)^16^ rather than an empirical background was used to estimate the background. CAMS v21r1 only used ground-based measurements that are mostly located in the marine boundary layer. The estimated background from three approaches agrees well with observations that had minimal influences by emissions (Figures S6 and S7), i.e., observations made at coastal areas primarily influenced by marine air (e.g., at TGC in Texas coast in summer and at ESP in the west coast of Canada) and continental observations with little enhancements.

### 2.4 Reported Emissions and Their Uncertainties

The full posterior error covariance matrix (*V*) of the optimized grid-scale scaling factors (λ) of surface emissions for each inversion is calculated with eq 7

7 $$V=Q-QK^{T}{\left(KQK^{T}+R\right)}^{-1}KQ$$

The posterior error covariance matrix of the optimized emissions (*C*) for each inversion can be derived from *s*_p_^*T*^ *Vs*_p_. The error covariances for national and regional emissions were calculated by summing the corresponding rows and columns in *C* to account for cross error correlations in space and time. The final 1σ errors for our inversion ensemble are estimated based on errors from individual inversions and their ensemble spread described in Hu et al.^29^ In the results discussed below, we report 2σ errors at the 95% confidence interval unless specified otherwise.

## 3 Results and Discussion

### 3.1 Derived U.S. CH_4_ Emissions

The total net CH_4_ emissions from the contiguous U.S. (CONUS) in our two prior estimates are 38 Tg yr^–1^ (CT-CH4-2014) and 34 Tg yr^–1^ (GHGI2020 + nat) for 2015–2017, whereas our posterior emissions optimized by atmospheric observations were adjusted upward consistently across all scenarios using different transport models, prior emissions, and background estimations (Figure S8). This resulted in total net CH_4_ emissions of 50 ± 6 Tg yr^–1^ for 2015–2017, with estimates from HYSPLIT-NAMS/GFS at 51 ± 5 Tg yr^–1^ and from WRF-STILT at 48 ± 3 Tg yr^–1^ (Figure 1; Table S1). We focus on 2015–2017 and consider it as our reference period for subsequent discussions, because this is the period when footprints from two transport models are available. The upward adjustment in our inversions is most notable in the east, midwest, and central south regions, especially over Oklahoma and Texas (Figure S9). Our derived magnitude for the U.S. CH_4_ emissions agrees (within errors) with many other inverse estimates that were optimized against in situ observations^5,16–18^ and/or satellite-based remote sensing observations^5,14,19,40,41^ (Figure 1; Table S2). The exceptions are the CAMS version 21r1 global in situ inversion results, as well as the inversion results reported by Maasakkers et al.^40^ and Nesser et al.,^42^ which appear to be statistically lower. However, these estimates are still in the range of inverse estimates compiled by the Global Carbon Project—CH_4_.^5^ In addition to the magnitude, the spatial distribution of U.S. CH_4_ emissions derived from this study (Figure 2) is also similar to some other inverse results such as from CT-CH4-2023 and Miller et al.^18^ (Figure S9).

**Figure 1:**
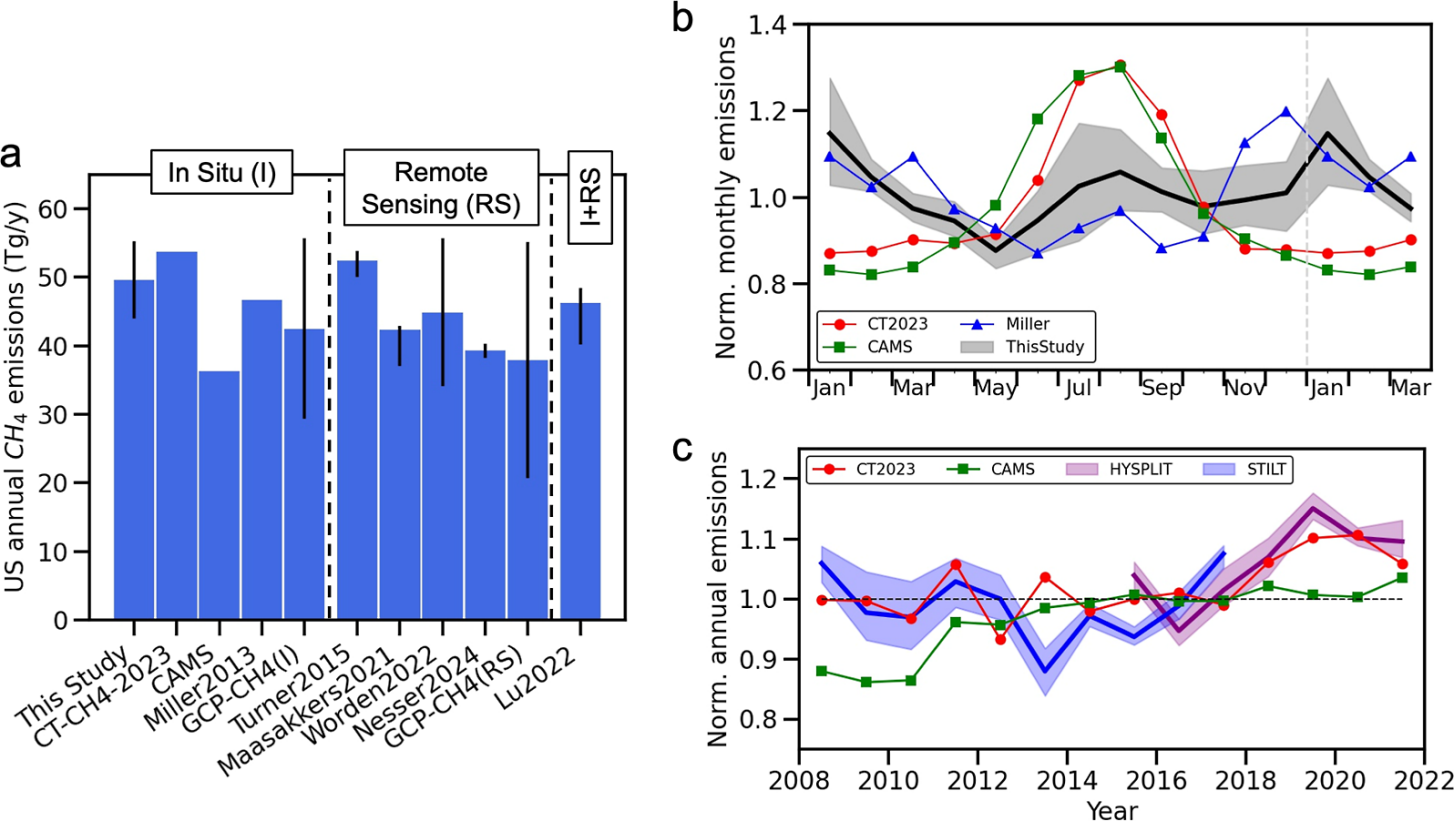
U.S. total net CH_4_ emissions derived from this study and from other inverse estimates. (a) Multi-year average annual CH_4_ emissions derived from this study and other studies. Errorbars indicate 2σ or the range reported by other studies. Note that some of the studies focused on a different time period from this study. The individual study periods are listed in Table S2. (b) Normalized multi-year average monthly CH_4_ emissions from this study (a black line with a gray shading denoting the 95% confidence interval), CT-CH4-2023 (red circles connected with a red line), and CAMS (green squares connected with a green line) between 2015 and 2017 and from Miller et al.^18^ (blue triangles connected with a blue line) for 2007–2008. (c) Trends in U.S. CH_4_ emissions derived from this study, CT-CH4-2023, and CAMS. Emissions are normalized by the average values in 2015–2017. Color shadings in panels (b,c) denote the 95% confidence interval.

**Figure 2:**
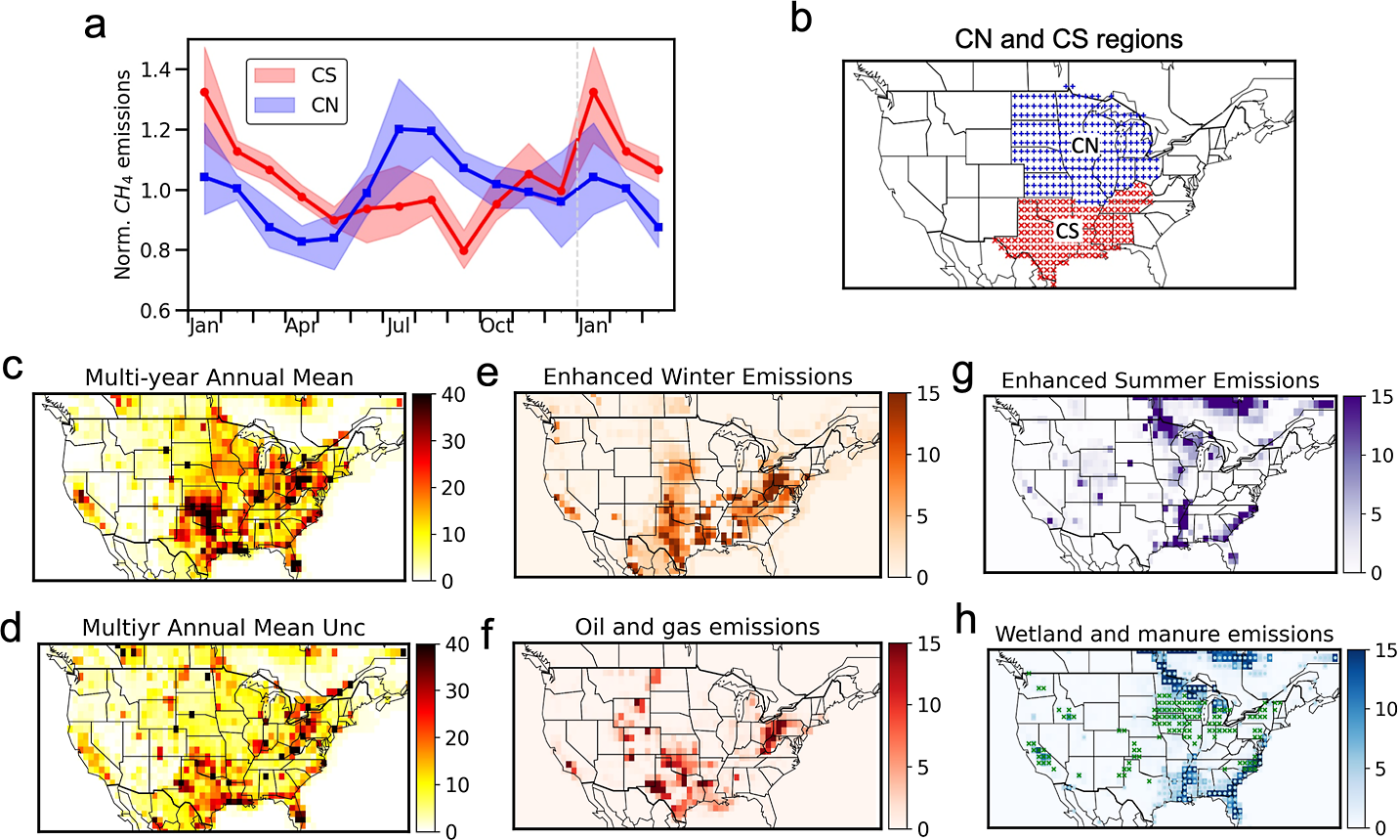
Seasonal variations of CH_4_ emissions derived for regions dominated by oil and gas production versus those dominated by wetlands and agriculture. (a) Normalized multi-year average seasonal cycle of CH_4_ from the central north (blue) and central south (red) regions of the U.S. between 2015 and 2017. (b) Definitions of central north (blue) and central south (red) regions. (c,d) Multi-year average annual emissions of CH_4_ in 2015–2017 and their associated 2σ uncertainties. (e,f) Areas with enhanced winter CH_4_ emissions (upper panel) and annual oil and gas CH_4_ emissions reported by Maasakkers et al. (2023) (lower panel). (g,h) Areas with enhanced summer CH_4_ emissions (upper panel) and annual CH_4_ emissions from wetland and manure management (lower panel). Wetland emissions are the average between the Kaplan^43^ and TEM^44^ models, whereas emissions from manure management are from Maasakkers et al. (2023). Grid cells with light blue dots indicate areas with wetland CH_4_ emissions, whereas grid cells with green crosses indicate areas with CH_4_ emissions from manure management. Panels (c–h) show emissions in nmol m^–2^ s^–1^.

Besides the magnitude and distribution of CH_4_, we further investigated trends in U.S. CH_4_ emissions from 2008 to 2021 in our posterior estimates. To avoid systematic differences between the two models causing erroneous trend interpretations, we normalized the annual emissions by the average emissions in our reference period (2015–2017) for each scenario. Our results show a roughly 10% increase from 2008 to 2021. This increase primarily occurred between 2016 and 2019, followed by a decline in emissions in 2020 and 2021 (Figures 1 and S8), which may be related to changes in emissions activities during and after the coronavirus disease 2019 (COVID-19) pandemic. Our derived trend is similar to the trend inferred from CT-CH4-2023 ^17^ (Figure 1), but differs from CAMS in the timing when the increase of CH_4_ emissions occurred (Figure 1). In addition, the trend inferred from this study aligns well with those reported in some other studies that focused on a subset of years. For example, trends in U.S. total CH_4_ emissions were reported to be 0.3–0.9% yr^–1^ between 2010 and 2017 by Maasakkers et al.^40^ and Lu et al.,^14^ while our results suggest it to be 0.01 ± 1.1% yr^–1^ for this period.

Among the various aspects we examined—emission magnitude, spatial distribution, and trend—the most striking finding is the distinct seasonality of U.S. CH_4_ emissions derived from the current study, which contrasts with both our prior emission estimates and previous studies. The two prior emissions considered in this analysis show higher CH_4_ emissions in summer than in winter (Figures S4 and S8). Emissions in the prior GHGI2020 + nat are 8% higher in summer than in winter, whereas emissions in the prior CT-CH4-2014 are 60% higher in summer than in winter (Figures S4 and S8). The higher summer emissions in both priors are primarily from wetlands and agriculture, specifically manure management according to Maasakkers et al.^36^ In contrast to the prior emissions, U.S. total net CH_4_ emissions derived from our posterior estimates do not show a strong summer peak; instead, they reveal two peaks: one in winter and one in summer (Figure 1; Table S2) with the winter peak similar or slightly stronger than the summer peak (Figure S3), depending on which transport model we use.

To place the seasonality derived from this study in the context of other inversion analyses, we analyzed monthly fluxes reported from CT-CH4-2023 and CAMS, both of which only used a subset of ground and aircraft measurements compared to this analysis. We also considered the regional inverse modeling result from Miller et al.^18^ CT-CH4-2023 and CAMS show similar seasonality as our prior estimates: both had emissions ∼40% higher in summer than in winter, whereas Miller et al.^18^ estimated U.S. CH_4_ emissions that showed a more similar seasonal cycle. In contrast to global models such as CT-CH4-2023 and CAMS, our regional model assimilates more observations. It also simulates atmospheric transport and performs inversions at much higher resolutions, potentially allowing a better representation of atmospheric transport^45–48^ and a reduction in model representation errors and aggregation errors.^49^ Furthermore, the previous national-scale inversion by Miller et al.^18^ did not include OH losses. If OH losses had been included, a seasonal cycle more similar to what is estimated here would be expected.

To identify regions driving the seasonality in overall U.S. CH_4_ emissions, we divided the CONUS into six regions (Figure S10) and analyzed the seasonal variations in regional emissions. Results indicate that CH_4_ emissions from the central north and central south regions show the most pronounced seasonality (in Tg yr^–1^), with their seasonal patterns being near opposite in phase (Figure 2). In the central south, CH_4_ emissions are higher in winter, while in the central north, they are higher in summer with a smaller secondary peak in winter (Figure 2; Table S3). This leads to two peaks in the overall seasonality in CH_4_ emissions from CONUS (Figure 1). Although we only plotted the average seasonality in 2015–2017 in Figures 1, 2, and S8, similar seasonal patterns in CH_4_ emissions in the central north and central south regions are shown in other years of this study, regardless of the transport model, background values, or prior emission used (Figure S11). However, the amplitudes of these seasonal cycles vary from year to year and between different transport models (Figures S3, S10, and S11). In addition to the central north and central south regions, the northeast region also shows a consistent seasonal pattern with emissions 41 (25–56) % higher in winter than in summer (Figure S12).

Besides analyzing seasonal variations in regional emissions, we also investigated the spatial distribution of this seasonality by subtracting summer emissions from winter emissions. The obtained results suggest that, in our posterior estimates, regions with enhanced winter emissions are primarily located in the oil and gas production regions, whereas in wetlands and areas with manure management, CH_4_ is more enhanced in summer (Figure 2). The seasonality in CH_4_ emissions from wetlands and manure management in the posterior estimates is overall consistent with the two prior emissions (Figure S4), whereas the enhanced winter emissions over the oil and gas regions are absent from the prior estimates (Figure S4) and are derived from the inverse modeling of atmospheric observations. Considering all the grid cells that are dominated by oil and gas emissions (defined as grid cells where oil and gas emissions accounted for more than 50% of their total prior CH_4_ emissions), we estimate that the amplitude of this seasonal cycle expressed as the ratio of excess emissions in winter relative to summer is 36 (21–50)%. Compared to this study, CT-CH4-2023 and CAMS had similar seasonality from wetlands and agriculture areas, but lacked seasonality over the oil and gas regions (Figure S13). Although inverse estimates by Miller et al.^18^ had enhanced winter CH_4_ emissions, there is no distinct spatial pattern indicating a specific source sector where the enhanced winter emissions were from (Figure S13).

Although seasonal changes in winds and other meteorological conditions can alter areas where our atmospheric measurements are sensitive to, the overall footprint patterns remain largely similar between winter and summer, with only minor regional variations (Figure S14). More importantly, in most oil and gas basins where we identified enhanced winter emissions, our atmospheric measurements exhibited strong sensitivities in both winter and summer, suggesting that significant biases due to seasonal meteorological changes are unlikely. Furthermore, we conducted an observing system simulation experiment (OSSE) to quantify potential biases in the estimated emissions due to gaps in our atmospheric observing system that resulted in lower sensitivities over certain regions such as over part of the Permian basin. In this experiment, we imposed a 50% seasonal cycle in the oil and gas CH_4_ emissions (peaking in Jan) and added non-oil and gas CH_4_ emissions from the prior GHGI2020 + nat to create “synthetic-true” emissions and their corresponding “synthetic observations” (see Supporting Information Text S6). We then conducted inversions with a priori (CT-CH4-2014) that had significantly different spatial distribution and an opposite seasonal cycle than the true emissions (Figure 3). Results suggest that, even with a defective prior, we can substantially correct the spatial and seasonal biases of the prior emissions (Figure 3) and derive a seasonal cycle that is very similar to the true emissions. The lower sensitivity over the Permian basin, for example, has not negatively affect the regional emission estimates much (Figure 3k). Note that, we also conducted an inversion with an alternative prior (GHGI2020 + nat) that had the same spatial distribution as true emissions, but lacked the seasonal cycle in the oil and gas emissions. With this better a priori, the derived posterior emissions are near identical to the truth on national and regional scales (Figure S15).

**Figure 3:**
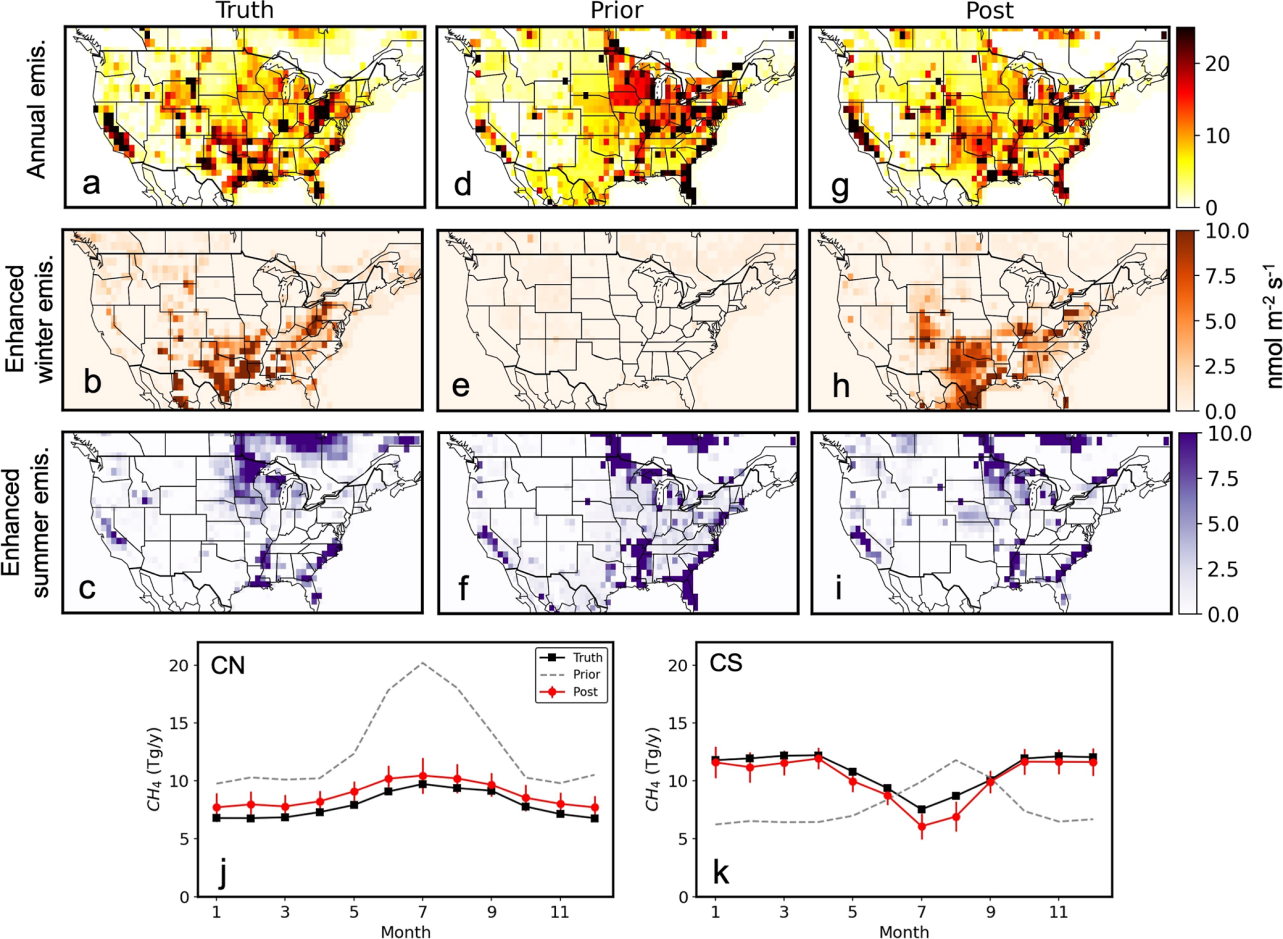
Synthetic-true, prior, posterior CH_4_ emissions from OSSEs. (a) Synthetic-true annual emissions averaged between 2015 and 2017. (b) Areas with enhanced winter emissions in the synthetic-true emissions. (c) Areas with enhanced summer emissions in the synthetic-true emissions. (d–f) The same as panels (a–c) but for prior emissions. (g–i) The same as panels (a–c) but for posterior emissions. (j–k) Multi-year average monthly emissions for CN and CS regions from synthetic-true emissions, prior, and posterior emissions between 2015 and 2017. The definitions of the CN and CS regions are shown in Figure 2.

Lastly, the seasonal cycle in OH concentrations can also affect the seasonal amplitude of CH_4_ emissions derived on national and regional scales. To assess the potential biases related to OH chemistry, we compared the OH field used in this study versus that from the GEOS-Chem model^19^ (Figure S16). Although there was about 20% difference in the OH concentrations between these two products, their seasonal amplitude of OH is similar. Inversions with the GEOS-Chem OH yielded a seasonal cycle that is only 1% different from results discussed above on the national scale (Figure S16).

### 3.2 Derived Seasonal CH_4_ Emissions Consistent with Atmospheric CH_4_ Observations

Although the seasonality we derived for U.S. CH_4_ emissions has not been previously reported, it aligns well with the temporal variability and spatial distribution of the seasonality in atmospheric CH_4_ observations made at sites across the U.S. At sites strongly influenced by oil and gas activities, such as SGP in the Southern Great Plains, Oklahoma and WKT in Moody, Texas, atmospheric CH_4_ mole fractions are more elevated in winter (Figure S17), whereas at sites dominated by wetland emissions, such as LEF in Park Falls, Wisconsin, atmospheric CH_4_ mole fractions are higher in summer (Figure S17). It is noteworthy that seasonal variations in OH chemistry can also result in higher atmospheric concentrations in winter than in summer,^50^ but the magnitude of seasonal variations observed at SGP and WKT is substantially larger than that observed at marine boundary layer sites (see Figure S18 and East et al.^50^), indicating the seasonal enhancements at SGP and WKT have to be explained by factors beyond OH chemistry. While seasonal variations in planetary boundary layer depths and winds could also affect seasonal patterns of atmospheric CH_4_ enhancements, the observed seasonal changes at SGP, WKT, and LEF were likely driven primarily by seasonal emissions specific to these areas. This can be supported by the fact that the observed seasonal enhancements at SGP, WKT, and LEF can only be explained when accounting for the seasonality of regional emissions, in addition to seasonal meteorological changes. Specifically, when we convolved prior emissions (i.e., prior GHG2020 + nat with little seasonal changes) with footprints, the simulated mole fractions can barely match the observed seasonal enhancements at these sites (Figure S18). In contrast, the simulated mole fractions using our posterior emissions, which include stronger seasonality, can replicate the observations fairly well (Figure S18).

Secondly, the seasonality in atmospheric CH_4_ observations and our derived seasonality in CH_4_ emissions are unique for CH_4_ compared to other trace gases that do not share the common emission sources. For example, the seasonal changes observed in CH_4_ at SGP, WKT, and LEF were not observed in the measured enhancements of other trace gases such as N_2_O and HFC-134a (Figure S17) that are not largely emitted from oil and gas activities or wetlands. For N_2_O and HFC-134a, whose atmospheric mole fractions are more enhanced in spring or summer at these locations (Figure S17), we derived posterior emissions that are higher in late spring and early summer (for N_2_O) or in summer (for HFC-134a) (Figure S19). In cases where seasonal changes in observed atmospheric enhancements were primarily caused by meteorology, we noted a common seasonal pattern in the atmospheric observations of multiple trace gases; e.g., at two sites in California (STR and WGC, Figure S1), we observed higher enhancements in atmospheric CH_4_ during winter, while a similar seasonal pattern was also observed in the enhancements of atmospheric N_2_O and HFC-134a (Figure S17). These observations suggest that the seasonal atmospheric CH_4_ enhancements at these two locations were caused largely by coastal winds in California that change directions seasonally.

Lastly, evaluation of our derived emissions against atmospheric data, both used in and independent of the inversions, suggests the simulated mole fractions agree well with atmospheric observations (Figures S18, S20–S23). The strong agreement between our simulations and aircraft profiles not only indicates that our estimated emissions well represent emission-related surface gradients in the atmosphere, but also that there are no significant biases in the vertical mixing of our transport models (Figures S21 and S22). In contrast, simulations from CT-CH4-2023 and CAMS, which lacked enhanced winter emissions and exhibited stronger summer emissions than our estimates show poorer agreement with observations, particularly at sites near oil and gas production regions (Figure S23). For instance, at SGP, the correlation (Pearson’s *r*) between our posterior simulated mole fractions and observations is 0.88 with a root mean square error (RMSE) of 29 ppb, whereas the correlations between posterior simulations and observations are 0.29 for CT-CH4-2023 and 0.31 for CAMS with RMSEs of 100 ppb (CT-CH4-2024) and 104 ppb (CAMS).

### 3.3 Derived Seasonal Emissions of CH_4_ supported by Other Trace Gas Observations

Our derived seasonality in U.S. CH_4_ emissions is not only consistent with the atmospheric CH_4_ observations across the U.S., but is also supported by other trace gas measurements made over this region that reflect emission processes. Ethane (C_2_H_6_) and propane (C_3_H_8_), minor components of raw natural gas, are often used to distinguish and quantify fossil-fuel-related contributions to CH_4_.^18,51–54^ At NOAA, we measure C_2_H_6_ and C_3_H_8_ from whole-air flasks collected from towers and aircraft. Inverse modeling of C_3_H_8_ (see Supporting Information Text S7) suggests that C_3_H_8_ is primarily emitted from oil and gas regions across the U.S. (Figure 4a). Our results also reveal a seasonal pattern in C_3_H_8_ emissions that is similar to CH_4_ over the oil and gas regions (Figure 4b,c). Notably, the derived wintertime C_3_H_8_ emissions are about 30–40% higher than the summertime emissions, close to the seasonal amplitude [36 (21–50)%] of CH_4_ emissions inferred from areas dominated by oil and gas activities. Although we did not conduct C_2_H_6_ inversions in this study, atmospheric C_2_H_6_ observations made over the U.S. show a similar seasonal cycle as C_3_H_8_ with a correlation of 0.96 (Pearson’s r) (Figures S24 and S25), indicating that, if an inversion was performed, a similar seasonal cycle would be expected to be derived for C_2_H_6_. Indeed, inverse modeling of C_2_H_6_ based on the atmospheric carbon and transport-America (ACT-America) campaign data^55^ (Figure 4d) also shows enhanced wintertime emissions than summertime.

**Figure 4:**
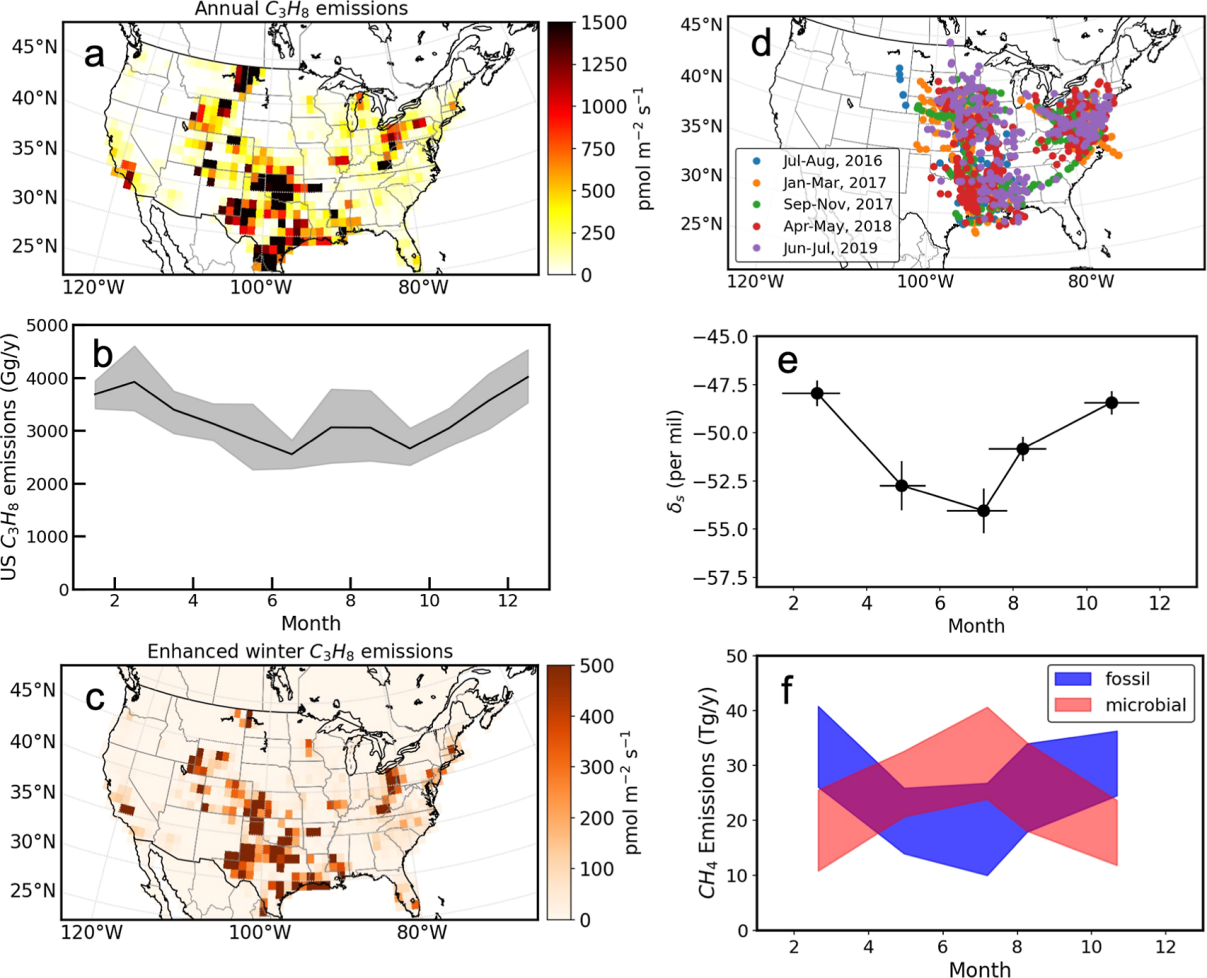
Seasonal cycle in U.S. C_3_H_8_ emissions and fossil fuel CH_4_ emissions estimated from atmospheric inversions of C_3_H_8_ or δ^13^CH_4_ observations. (a,b) Multi-year average annual and monthly C_3_H_8_ emissions between 2008 and 2015. (c) Areas with enhanced winter C_3_H_8_ emissions relative to summer. (d) Locations of δ^13^CH_4_ samples collected from ACT-America campaigns. (e) δ^13^CH_4_ source signatures derived from ACT-America campaigns. (f) Estimated U.S. fossil fuel and microbial CH_4_ emissions.

Besides C_2_H_6_ and C_3_H_8_, atmospheric δ^13^CH_4_ (the ^13^C/^12^C ratio of CH_4_ in a measured sample relative to the reference) was suggested to be useful to provide source information for CH_4_.^56^ This is because microbial, fossil fuel, and biomass burning emissions of CH_4_ have distinct δ^13^CH_4_ source signatures, which are −64 (±4, 1σ)‰, −44 (±4, 1σ)‰, and −25 (±7, 1σ)‰ over the U.S. Given that biomass burning emissions of CH_4_ are negligible over the CONUS (∼0.3 Tg yr^–1^ based on the Global Fire Emissions Database^57^), atmospheric δ^13^CH_4_ signatures are primarily influenced by fossil fuel and microbial emissions of CH_4_ over this region. At NOAA, δ^13^CH_4_ is not often measured in the whole-air flasks collected from tower and aircraft sites in the U.S. However, there were δ^13^CH_4_ measurements made from the ACT-America campaigns^58^ that covered different months between 2016 and 2019 in the eastern and mid-U.S. (Figure 4d). The isotopic source signatures inferred from CH_4_ and δ^13^CH_4_ measurements from ACT-America are heavier in the fall and winter campaigns (−48‰ to −49‰) and lighter in spring and summer campaigns (−52‰ to −58‰) (Figure 4e). The heavier isotopic source signatures in winter and fall indicate a larger fraction of fossil fuel emissions in these two seasons than spring and summer. By combining the seasonal isotopic source signatures from ACT-America with CH_4_ emissions derived from inversions (see Supporting Information Text S8), we estimate that U.S. fossil fuel CH_4_ emissions are 26 (19–32) Tg yr^–1^ averaged across all 5 campaigns, 35 (26–41) Tg yr^–1^ during the winter campaign, and 23 (14–30) Tg yr^–1^ averaged between the two summer campaigns (Figure 4f). The net microbial CH_4_ emissions were estimated to be 24 (17–31) Tg yr^–1^ averaged across 5 campaigns, 17 (11–26) Tg yr^–1^ during the winter campaign, and 28 (21–37) Tg yr^–1^ averaged between summer campaigns (Figure 4f). The seasonal amplitude in fossil fuel CH_4_ emissions, derived from δ^13^CH_4_ observations, is 46 (27–130)% higher in winter than in summer (Figure 4f). The estimated seasonal amplitude agrees within errors with the seasonal amplitudes derived from CH_4_ or C_3_H_8_ inversions.

### 3.4 Potential Causes of Seasonal Oil and Gas CH_4_ Emissions

The presence of a seasonal cycle in CH_4_ emissions from oil and gas activities has not previously been reported on the U.S. national scale and is currently absent from emission inventories (Figure S26). However, a similar seasonal cycle from oil and gas CH_4_ emissions as identified here has been reported from urban areas, such as over Boston,^22^ Washington, DC, and Baltimore,^23^ and Los Angeles^59,60^ with winter emissions 40–100% higher than summer emissions. All of these urban studies attributed such a seasonality to increased CH_4_ leakage from natural gas consumption due to increased wintertime heating demand. This process has likely contributed to the seasonal cycle estimated in the present study on the national scale. Elevated winter emissions are indeed shown near some of these urban areas and also derived for the populated northeast US and parts of California (Figures 2 and S12). However, the overall urban contribution to our derived national-scale seasonal cycle does not appear prominently in the seasonality map shown in Figure 2. In contrast, the enhanced winter CH_4_ emissions are primarily located near or in the oil and natural gas production regions, indicating that they are likely associated with drilling or production-related activities, despite there being little seasonality in reported natural gas production rates.^61^ Moreover, it is also likely that increased CH_4_ emissions during winter may result from higher leakages from natural gas gathering and transmission systems due to increased consumption for winter heating, which shows a similar seasonal pattern that peaks in January (Figure S27). Besides natural gas consumption, there are also strong seasonal cycles in reported underground storage quantities of natural gas (Figure S27). Underground storage of natural gas typically reaches its peak capacity in November and the lowest capacity in March. Since its timing differs from the seasonality of oil and gas CH_4_ emissions derived from this study (Figure 2), we do not believe that leakage from underground storage is a major cause of enhanced winter CH_4_ emissions in the oil and gas production regions. However, the exact processes driving this seasonality remain unclear based on the insights learned from atmospheric tracer gas observations discussed above. Identifying the specific processes causing the higher wintertime oil and gas CH_4_ emissions could improve industries’ operational efficiency, minimize associated economic losses, and furthermore, reduce the influence of the oil and gas CH_4_ emissions on atmospheric CH_4_ levels.

## Supplementary Material



## Data Availability

Posterior CH_4_ emissions estimated from this analysis are available at https://doi.org/10.15138/zmd2-cy30. NOAA’s CH_4_, C_3_H_8_, N_2_O, and HFC-134a data are publicly available and can be downloaded from https://gml.noaa.gov/dv/data/. The ACT-America data can be accessed from 10.3334/ORNLDAAC/1593. WRF-STILT footprints are available at https://gml.noaa.gov/aftp/products/carbontracker/lagrange/footprints/ctl-na-v1.1/. HYSPLIT-NAMS/GFS footprints computed from this study are available at https://gml.noaa.gov/aftp/products/carbontracker/lagrange/footprints/hysplit496-nams/flask. For other questions related to data used or produced from this paper, please contact the corresponding author: Dr. Lei Hu (lei.hu@noaa.gov and leihutx@gmail.com).

## Notes

Additional details on methods for calculation of footprints, inverse modeling of CH_4_, C_3_H_8_, N_2_O, and HFC-134a, observing system simulation experiments, and estimation of isotopic source signatures and sector-based emissions using ACT-America data are provided in the supplement. Additional supplemental figures and tables are also included in the supplement (PDF)
