# Supplementary material for: An Unexpected Seasonal Cycle in U.S. Oil and Gas Methane Emissions
Source: Environ Sci Technol. 2025 May 14;59(20):9968–79. doi: 10.1021/acs.est.4c14090 (PMC12120981; doi:10.1021/acs.est.4c14090)
Supplement: Supplementary file 1 [file es4c14090_si_001.pdf]

Supporting Information for

"An unexpected seasonal cycle in U.S. oil and gas methane emissions"

Lei Hu[1]*, Arlyn E. Andrews[1], Stephen A. Montzka[1], Scot M. Miller[2], Lori Bruhwiler[1], Youmi Oh[3], Colm Sweeney[1], John B. Miller[1], Kathryn McKain[1], Sergio Ibarra Espinosa[3], Kenneth Davis[4,5], Natasha Miles[4,1], Marikate Mountain[6], Xin Lan[3], Andy Crotwell[3], Monica Madronich[3], Thomas Mefford[3], Sylvia Michel[7], Sander Houwelling[8]

[1]Global Monitoring Laboratory, US National Oceanic and Atmospheric Administration, Boulder, CO, 80305, USA
[2]Department of Environmental Health and Engineering, Johns Hopkins University, MD, 21218, USA
[3]Cooperative Institute for Research in Environmental Sciences, University of Colorado – Boulder, Boulder, CO, 80309, USA
[4]Department of Meteorology and Atmospheric Science, The Pennsylvania State University, University Park, PA 16802, USA
[5]Earth and Environmental Systems Institute, The Pennsylvania State University, University Park, PA,16802, USA
[6]Atmospheric and Environmental Research Inc., Lexington, MA 02421, USA
[7]Institute for Arctic and Alpine Research, University of Colorado-Boulder, Boulder, CO, 80309, USA
[8]Department of Earth Sciences, Vrije Universiteit Amsterdam, 1081 HV Amsterdam, the Netherlands

*Correspondence to: lei.hu@noaa.gov and leihutx@gmail.com

This file contains:

Supplementary Text S1 – S8
Tables S1 – S5
Figures S1 – S31

Supplementary Text.

S1. Calculation of footprints (*H*)

H was computed from two Lagrangian atmospheric transport models driven by different meteorological fields: the Hybrid Single-Particle Lagrangian Integrated Trajectory model [1] driven by the North American Mesoscale Forecast System nested with the Global Forest System (HYSPLIT-NAMS/GFS) and the Stochastic Time-Inverted Lagrangian Transport model driven by the Weather Research and Forecasting (WRF-STILT) model. Uncertainties generated from a formal inversion do not include uncertainties related to systematic biases of transport. Using two transport models enables us to include such uncertainties in the final results.

The NAMS meteorology contains hourly 12-kilometer forecasts at 40 sigma-pressure levels [2] covering the majority of the North American continent except for Alaska and the lower part of Mexico. We used the GFS 0.5°-archive before June 2019 and 0.25°-archive after. The GFS archives contain global 3 hourly outputs at 55 sigma pressure levels. The WRF modeled fields had 10-km spatial resolution below 55°N over temperate North America and 30- to 40-km spatial resolution above 55°N during 2007–2010. After 2010, the high-resolution (10 km) domain was extended to 70°N, covering most of the North American continent except for Alaska. The vertical resolution in the WRF runs was extended from 30 levels in 2007 – 2008 to 41 levels in the later years. Both HYSPLIT-NAMS/GFS and WRF-STILT models were run backward in time with 500 particles for 10 days to calculate footprints at 1° × 1° × 1 hour resolution.

S2. Setting up the state vector (λ)

We optimized weekly scaling factors (λ) for CH_4 emissions at 1° × 1° over the U.S., Canada, an upper part of Mexico, and their adjacent oceanic grid cells (Fig. S1). To solve for λ, we divided all the observations into multiple batches and conducted a direct matrix inversion for each batch using the algorithm developed by Yadav and Michalak [3]. In each batch, we included an entire year along with two weeks before and after the year. To minimize the end effects on the reported emissions, we excluded the solutions for the first and last two weeks [4]. We also tested extending the batch window to five weeks before and after the year, but found no significant differences in derived emissions compared to those obtained with a shorter batch window.

S3. Estimation of error covariance matrices (R and Q)

R and *Q* are the model-data mismatch and prior emission error covariance matrices. The values specified in *R* and *Q* are critical for determining the appropriate weights between atmospheric observations and prior emissions in the final solution. Similar to inverse modeling of other gases [5, 6], we assume that observations included in the inversion are independent among each other and that R is a diagonal matrix that contains seasonally- and interannually- varying values at individual sites [4, 7]. We also assume that errors in prior emissions (Q) vary seasonally and interannually and are correlated in space and time; but their correlations decays exponentially [4]. Both R and Q were derived objectively with maximum likelihood estimation [7, 8].

S4. Estimating the impact of OH losses on inversely modeled CH_4 emissions

To quantify the impact of CH_4 losses to OH, we used monthly 4D OH from CT-CH4-2023, which is the climatology OH product from Spivakovsky et al. [9] and optimized against global atmospheric

methyl chloroform observations [10]. For each observation, we calculated $\chi_{bg} \exp\left(\int_0^{t1}(-\kappa\chi_{OH}dt)\right)$ based on the 500 air back-trajectories computed from HYSPLIT-NAMS/GFS and WRF-STILT. This calculation utilizes the time intervals of the 500 particles from their entry into the model domain to their arrival at the measurement location. The chemical reaction rate constant (κ) is calculated by Eq. S1:

$$\kappa = Ae^{-\frac{E}{RT}} \quad \text{(S1)}$$

where A = 2.45 × 10^{-12} (molecule cm^{-3})$^{-1}$ s^{-1} [11]; E/R = 1775 (unit: K) [11]; and T is the Kelvin temperature at each time step from the HYSPLIT-NAMS/GFS or WRF-STILT transport model. We calculated an OH-corrected background (e.g., Figs. S6 – S7) based on each of the 500 back-trajectories and averaged them to obtain an OH-corrected background estimate for each observation.

The correction of chemical losses on new emissions is equivalent to applying a fractional loss into the calculated footprints, $H \exp\left(\int_0^{t2}(-\kappa\chi_{OH}dt)\right)$. In HYSPLIT-NAMS/GFS or WRF-STILT, the magnitude of footprints are calculated based on the density of air particles (normalized by the total number of particles in the model run) in the planetary boundary layer at each grid cell and each time step [12]. To calculate the reduction of footprints or sensitivity related to chemical losses to OH, we first identified grid cells with non-zero footprints at each time step. We used their corresponding back-trajectories to estimate the fractional loss from the time when particles were in the planetary boundary layer to the time of the measurement.

S5. Constructing the 4D empirical background

The 4D empirical background field was constructed from two Marine Boundary Layer References (MBLRs) over the Pacific and Atlantic Ocean basins (https://gml.noaa.gov/ccgg/mbl/mbl.html) and four Free Troposphere References (FTRs) at 2 – 5 km, 5 – 6 km, 6 – 7 km, and 7 – 8 km. Each MBLR or FTR is a smoothed representation of background CH_4 mole fractions in a clean remote atmosphere either in the boundary layer or at a specified altitude in the free troposphere. The two MBLRs were constructed based on atmospheric measurements made in the marine boundary layer over the Pacific or Atlantic Ocean basin (Fig. S5) and a curve-fitting and data extension method [13]. FTRs were created with the same curve-fitting and data extension method, but based on aircraft measurements, many of which are located in North America (Fig. S5). To ensure the selected atmospheric observations for constructing FTRs were not strongly influenced by North American emissions, we only used observations that contain low sensitivity to surface emissions, *i.e.*, the summed 10-day footprints over North America were $\leq$ 1 ppb (nmol m^{-2} s^{-1})$^{-1}$ [5] for observations made at 2 – 5 km and nearly all observations made at 5 km and above. With the constructed MBLRs and FTRs, we created a 4D background field by interpolating their mole fractions every kilometer in altitude and every 5 degrees in both longitude and latitude every week.

S6. Observing system simulation experiments for CH_4

We conducted an observing system simulation experiment to assess the ability of our inversion system and flask-air sampling network in estimating seasonal variations of oil and gas CH_4

emissions. In this experiment, we used the oil and gas emissions from Maasakkers et al. [14] and imposed a 50% seasonal cycle that peaked in January. We then added other non-oil and gas emissions to create monthly gridded "synthetic-true" emissions. The non-oil and gas emissions are the same ones used in the prior GHGI2020+nat. We then convolved the synthetic-true emissions with the simulated WRF-STILT transport to generate "synthetic observations" that have the same sampling times and locations as those observations used in real-data inversions. These observations were further perturbed by adding 0.1-ppb Gaussian noises. With these synthetic observations, we conducted inversions with the two priors considered in our real-data inversions, i.e., CT-CH4-2014 and GHGI2020+nat, and the same framework described in the Methods section in the main text.

S7. Inverse modeling of C_3H_8, N_2O, and HFC-134a

Inverse modeling of C_3H_8 was performed with a similar modeling framework as CH_4 as described in the Methods section in the main text and with a similar suite of atmospheric whole-air flask measurements made by NOAA GML. Like CH_4, atmospheric C_3H_8 also shows stronger enhancements in winter than in summer, especially at sites within or near oil and gas production regions (Fig. S24). We conducted multiple inverse modeling runs to test the sensitivity of the posterior emissions to model setups (i.e., Bayesian versus geostatistical inversions), prior emissions, and OH losses (Fig. S28).

In a Bayesian inverse modeling framework, we considered four different prior emissions (Fig. S28a, c, d, e). We constructed the first a priori (Fig. S28a) based on the spatial distributions of oil production [15], natural gas production [15], and population density [16]. We evaluated different combinations of these three datasets based on Bayesian Information Criterion (BIC) [17]. The BIC score is the lowest when considering all three datasets together (Table S4), suggesting the combination of all three datasets together best explains the observed atmospheric variability of C_3H_8 compared to using any one or two datasets without overfitting. We then used the least square method to calculate optimal scaling factors of each dataset by minimizing the sum squared errors of simulated mole fractions relative to atmospheric observations on a monthly basis. The first a priori was then constructed as the weighted sum of these three geospatial datasets based on these calculated monthly scaling factors. The second – fourth a priori has constant emission rates in space and time, which we call them as "flat" priors. The differences among these three priors are their total emission magnitudes over the contiguous U.S. (CONUS) (0.4 Tg yr^{-1} or 4 Tg yr^{-1}) or whether there are emissions in grid cells over the ocean. We set up inversions by solving for additive corrections (Fig. S28h, l) or scaling factors of prior emissions at 1° x 1° x monthly resolution (Fig. S28g, I, j, n, o).

Besides Bayesian inversions, we also considered a geostatistical inversion framework [17, 18] for estimating US C_3H_8 emissions at 1° x 1° x monthly resolution. Although the derived spatial distribution of the posterior emission had some dependence on the prior distribution, all of them consistently show that C_3H_8 was primarily emitted from oil and gas regions (Fig. S28) and that there were enhanced emissions in winter than in summer (Figs. 4 and S29). Note that we included the OH corrections on both estimated backgrounds and footprints in C_3H_8 inversions. Although we did not quantify the impacts of OH losses on the estimated backgrounds and footprints

separately, we expect its impact on the footprints is much stronger than that for CH_4 due to its shorter atmospheric lifetime.

Inverse modeling of N_2O and HFC-134a was performed with the same transport and inverse modeling framework as CH_4 as described in the Methods section in the main text, except that we did not consider their atmospheric losses. N_2O is primarily degraded in the atmosphere via stratospheric photolysis and there are no significant losses in the troposphere [19]. Thus, no chemical losses were included in our inverse modeling of N_2O. For HFC-134a, it is also degraded via reaction with tropospheric OH and it has a longer atmospheric lifetime than CH_4, i.e., 14 years [20]. Given a longer atmospheric lifetime, and more importantly, the relatively larger enhancement versus background ratios for HFC-134a (about 6 times greater than CH_4) over the CONUS, the relative impacts of OH losses on the derived HFC-134a emissions are expected to be much smaller than that of CH_4. If we consider the impact of OH losses on derived emissions of HFC134a, a slightly stronger seasonal cycle would be expected, with even more pronounced emissions in summer (Fig. S19). Dedicated description on inverse modeling of HFC-134a was described in Hu et al. [4].

S8. Estimating isotopic source signatures and sector-based emissions using ACT-America data

The isotopic signatures (δ_s) during the ACT-America campaigns were estimated from the slope using Eq. S2 [21]:

$$\delta^{13}CH_4\chi_{obs} - \delta^{13}CH_{4,bg}\chi_{bg} = \delta_s(\chi_{obs} - \chi_{bg}) \quad \text{(S2)}$$

where $\delta^{13}CH_4$ and χ_{obs} represent observed $\delta^{13}CH_4$ and CH_4 mole fractions measured in whole-air flask samples collected below 1 km asl from each campaign; $\delta^{13}CH_{4,bg}$ and χ_{bg} represent their corresponding background values, which were estimated from the mean values among the samples collected between 3 and 6 km asl from each campaign. δ_s was then obtained by performing a linear regression between $\delta^{13}CH_4\chi_{obs} - \delta^{13}CH_{4,bg}\chi_{bg}$ and $\chi_{obs} - \chi_{bg}$.

The estimated signatures obtained from individual ACT-America campaigns are representative of flux-weighted $\delta^{13}CH_4$ signatures (δ_s) influenced by both sources and sinks. Therefore, we can write the following equations:

$$\delta_{ff}F_{ff} + \delta_{mic}F_{mic} + \delta_{bb}F_{bb} + \delta_{OH}F_{OH} + \delta_{soil}F_{soil} = \delta_sF_{nf} \quad \text{(S3)}$$

$$F_{ff} + F_{mic} + F_{bb} + F_{OH} + F_{soil} = F_{nf} \quad \text{(S4)}$$

where, δ_{ff}, δ_{mic}, and δ_{bb} represent flux-weighted source signatures for U.S. fossil fuel CH_4 emissions (F_{ff}), U.S. microbial CH_4 emissions (F_{mic}), and U.S. biomass burning CH_4 emissions (F_{bb}). δ_{OH} and δ_{soil} denote the isotopic signatures of CH_4 sinks due to reaction with OH and uptake by soil over the U.S. F_{OH} and F_{soil} represent negative CH_4 fluxes related to OH and soil sinks. F_{nf} are the net total fluxes from all the sources and sinks. In Eqs. S3 and S4, all the parameters (except for F_{ff} and F_{mic}) were from the existing inventories, literature, or inversion results described below. We then solved for F_{ff} and F_{mic}.

S8.1. Estimating U.S. average $\boldsymbol{\delta_{ff}}$, $\boldsymbol{\delta_{mic}}$, and $\boldsymbol{\delta_{bb}}$

δ_{ff} was estimated by the U.S. average source signatures of coal, oil and gas, and other industrial activities, and weighted by their relative fluxes in the fossil fuel sector (Table S5). The U.S. average

source signatures of coal, oil and gas, and other industrial activities were derived based on the gridded source signatures compiled by Sherwood et al. [22]. Grid-scale source signatures were weighted based on the contributions of each grid flux to the total U.S. CH_4 emissions within each category to calculate a U.S. flux-weighted source signature for coal, oil and gas, or other industrial activities. Grid-scale 1σ uncertainties for the source signatures in coal was taken from the median uncertainty (~9 ‰) described in Lan et al. [23]. Grid-scale uncertainties for the source signatures in oil and gas was assumed to be 10% (~4 ‰), as more source signatures were available in this category [22]. Little is known on the uncertainty in the source signatures of other industrial activities, which we assumed to be 20% (1σ) on a grid-scale basis. The average source signatures from these sub-categories were then weighted by their emissions relative to the total U.S. fossil fuel emissions to estimate a U.S. averaged fossil fuel source signature. The weights for the subcategories were estimated based on the sector emissions from Maasakkers et al. [14] (coal, and oil and gas) and Oh et al. [24] (other industrial activities) (Table S5).

δ_{mic} was estimated by the U.S. average source signatures of wetland, ruminants, rice, waste, termite, and wild animals (Table S5). Grid-scale source signatures for wetland are from Oh et al.[25], assuming their 1σ uncertainties were 2.4 ‰ according to Lan et al. [23]. Source signatures for ruminants, rice, waste, termite and wild animals are from Lan et al. [23]. Spatial distributions of these source signatures were poorly known. There was only one value for the entire globe in each of these categories. Their grid-scale uncertainty was assumed to be 20% in this study, but could be even larger. The flux weight for each subcategory was estimated based on the combined emission information from the Kaplan model, Maasakkers et al. [14], and Oh et al. [24] (Table S5). An alternative set of weights were also considered by using wetland emissions from the TEM model [25].

δ_{bb} was from Lan et al. [23] for the CONUS region (Table S5). The calculated flux-weighted U.S. source signatures of δ_{ff}, δ_{mic}, and δ_{bb} are shown in Fig. S30.

S8.2. Estimating F_{nf}, F_{bb}, F_{OH}, and F_{soil}

F_{nf} was estimated from the monthly net surface emissions derived from the CH_4 inversions minus CH_4 losses related to OH (F_{OH}). Monthly F_{OH} was estimated from the difference between inversion-derived net surface CH_4 emissions with and without consideration of OH losses (Fig. S3). F_{soil} was estimated based on monthly CH_4 soil uptake simulated by the TEM model [25]. F_{bb} is estimated by monthly CH_4 emissions from the GFED database.

S8.3. Estimating δ_{OH} and δ_{soil}

The molar ratio of $^{13}CH_4/^{12}CH_4$ in a CH_4 sink (R_{sink}, sink = OH or soil) can be expressed as:

$$R_{sink} = R_{atm}\alpha_{sink} \quad \text{(S5)}$$

where R_{atm} denotes the $^{13}CH_4/^{12}CH_4$ ratio in the atmosphere. α_{sink} denotes the isotopic fractionation rate of a sink, which is defined as $\frac{k^{13}}{k^{12}}$, where k^{13} or k^{12} denotes the kinetic reaction rate $^{13}CH_4$ or $^{12}CH_4$ with OH or in the soil. Given the isotopic signature of a sink (δ_{sink}) or the atmosphere (δ_{atm}) can be expressed by their isotopic ratios (Eqs. S6 and S7),

$$\delta_{sink} = \frac{R_{sink}}{R_{PBD}} - 1 \quad \text{(S6)}$$

$$\delta_{atm} = \frac{R_{atm}}{R_{PBD}} - 1 \quad \text{(S7)}$$

where R_{PBD} denotes the $^{13}CH_4/^{12}CH_4$ ratio in a fossil belemnite from the Pee Dee Formation (PDB), we can express δ_{sink} as:

$$\delta_{sink} = (\delta_{atm} + 1)\alpha_{sink} + 1 \quad \text{(S8)}$$

Therefore, we can estimate δ_{OH} and δ_{soil} based on their isotopic fractionation rates, $\alpha_{OH} = 0.9961$ [26] and $\alpha_{soil} = 0.979$ [27], and δ_{atm} derived from observations made in the ACT campaigns.

S8.4. Estimating F_{ff} and F_{mic} and their uncertainties

With the parameters described in sections S8.1 – S8.3, we can solve for F_{ff} and F_{mic} with Eqs. S3 and S4. The uncertainties in F_{ff} and F_{mic} were estimated by considering both random uncertainties and systematic biases in the other parameters. Random uncertainties include those in the isotopic signatures in each of the sources described in Section S8.1, in the flux weights (assumed to be 30% for 1σ) due to our poor knowledge on emissions from different source categories, and in the observed isotopic signatures from atmospheric measurements (i.e., errors in the linear slopes).

Systematic biases were estimated by considering an alternative value α_{OH} (0.9946 [28]), two different wetland emissions (Table S5), and a potential bias in the soil uptake by TEM. Note that the soil uptake simulated by the TEM model is about twice larger than those reported by the Global Carbon Project – CH_4 [29] over the U.S. Therefore, we considered to scale the TEM soil uptake down by 50%. Besides these scenarios, we also tested the scenarios with systematic biases in F_{ff} and F_{mic}, where we adjusted their average source signatures upward by 4 ‰ (equivalent to their 1σ error).

All the results are shown in Fig. S31 with their associated uncertainties. Fig. 4f only shows the 32 best estimates (i.e., without the random uncertainties). Although the derived relative magnitudes between F_{ff} and F_{mic} are sensitive to different choices (Fig. S31), their estimated seasonal cycles are consistent among all the scenarios. They all indicate an enhancement in wintertime fossil fuel CH_4 emissions.

References for supporting information.

(1) Stein, A. F.; Draxler, R. R.; Rolph, G. D.; Stunder, B. J. B.; Cohen, M. D.; Ngan, F. NOAA's HYSPLIT atmospheric transport and dispersion modeling system. *Bulletin of the American Meteorological Society* **2015**. DOI: 10.1175/BAMS-D-14-00110.1 (acccessed 2015/05/21).

(2) NOAA Air Resources Laboratory. *NAMS Analysis Data Archive*. 2024. (accessed 2024 April 15).

(3) Yadav, V.; Michalak, A. M. Improving computational efficiency in large linear inverse problems: an example from carbon dioxide flux estimation. *Geosci. Model Dev.* **2013**, *6* (3), 583-590. DOI: 10.5194/gmd-6-583-2013.

(4) Hu, L.; Montzka, S. A.; Miller, J. B.; Andrews, A. E.; Lehman, S. J.; Miller, B. R.; Thoning, K.; Sweeney, C.; Chen, H.; Godwin, D. S.; et al. U.S. emissions of HFC-134a derived for 2008–2012

from an extensive flask-air sampling network. *Journal of Geophysical Research: Atmospheres* **2015**, 2014JD022617. DOI: 10.1002/2014JD022617.
(5) Hu, L.; Montzka, S. A.; Lehman, S. J.; Godwin, D. S.; Miller, B. R.; Andrews, A. E.; Thoning, K.; Miller, J. B.; Sweeney, C.; Siso, C.; et al. Considerable contribution of the Montreal Protocol to declining greenhouse gas emissions from the United States. *Geophysical Research Letters* **2017**, *44* (15), 2017GL074388. DOI: 10.1002/2017GL074388.
(6) Hu, L.; Ottinger, D.; Bogle, S.; Montzka, S. A.; DeCola, P. L.; Dlugokencky, E.; Andrews, A.; Thoning, K.; Sweeney, C.; Dutton, G.; et al. Declining, seasonal-varying emissions of sulfur hexafluoride from the United States. *Atmos. Chem. Phys.* **2023**, *23* (2), 1437-1448. DOI: 10.5194/acp-23-1437-2023.
(7) Michalak, A. M.; Hirsch, A.; Bruhwiler, L.; Gurney, K. R.; Peters, W.; Tans, P. P. Maximum likelihood estimation of covariance parameters for Bayesian atmospheric trace gas surface flux inversions. *Journal of Geophysical Research: Atmospheres* **2005**, *110* (D24), D24107. DOI: 10.1029/2005jd005970.
(8) Hu, L.; Montzka, S. A.; Miller, J. B.; Andrews, A. E.; Lehman, S. J.; Miller, B. R.; Thoning, K.; Sweeney, C.; Chen, H.; Godwin, D. S.; et al. U.S. emissions of HFC-134a derived for 2008–2012 from an extensive flask-air sampling network. *Journal of Geophysical Research: Atmospheres* **2015**, *120* (2), 801-825. DOI: https://doi.org/10.1002/2014JD022617.
(9) Spivakovsky, C. M.; Logan, J. A.; Montzka, S. A.; Balkanski, Y. J.; Foreman-Fowler, M.; et al. Three-dimensional climatological distribution of tropospheric OH: Update and evaluation. *Journal of Geophysical Research: Atmospheres* **2000**, *105* (D7), 8931-8980. DOI: doi:10.1029/1999JD901006.
(10) Montzka, S. A.; Krol, M.; Dlugokencky, E.; Hall, B.; Jöckel, P.; Lelieveld, J. Small Interannual Variability of Global Atmospheric Hydroxyl. *Science* **2011**, *331* (6013), 67-69. DOI: 10.1126/science.1197640.
(11) Burkholder, J. B.; Sander, S. P.; Abbatt, J. P. D.; Barker, J. R.; Cappa, C.; Counse, J. D.; Dibble, T. S.; Huie, R. E.; Kolb, C. E.; Kurylo, M. J.; et al. *Chemical kinetics and photochemical data for use in atmospheric studies, Evaluation No. 19*; JPL Publication 19-5, Jet Propulsion Laboratory, Pasadena, 2019. http://jpldataeval.jpl.nasa.gov.
(12) Lin, J. C.; Gerbig, C.; Wofsy, S. C.; Andrews, A. E.; Daube, B. C.; Davis, K. J.; Grainger, C. A. A near-field tool for simulating the upstream influence of atmospheric observations: The Stochastic Time-Inverted Lagrangian Transport (STILT) model. *Journal of Geophysical Research: Atmospheres* **2003**, *108* (D16), 4493. DOI: 10.1029/2002jd003161.
(13) Masarie, K. A.; Tans, P. P. Extension and integration of atmospheric carbon dioxide data into a globally consistent measurement record. *J. Geophys. Res.* **1995**, *100* (D6), 11593-11610. DOI: 10.1029/95jd00859.
(14) Maasakkers, J. D.; McDuffie, E. E.; Sulprizio, M. P.; Chen, C.; Schultz, M.; Brunelle, L.; Thrush, R.; Steller, J.; Sherry, C.; Jacob, D. J.; et al. A Gridded Inventory of Annual 2012–2018 U.S. Anthropogenic Methane Emissions. *Environmental Science & Technology* **2023**, *57* (43), 16276-16288. DOI: 10.1021/acs.est.3c05138.
(15) U.S. Department of Agriculture. County-level Oil and Gas Production in the United States. 2014.
(16) United States Census Bureau. County Population Estimates. 2013.

(17) Hu, L.; Montzka, S. A.; Miller, B. R.; Andrews, A. E.; Miller, J. B.; Lehman, S. J.; Sweeney, C.; Miller, S. M.; Thoning, K.; Siso, C.; et al. Continued emissions of carbon tetrachloride from the United States nearly two decades after its phaseout for dispersive uses. *Proceedings of the National Academy of Sciences* **2016**, *113* (11), 2880-2885. DOI: 10.1073/pnas.1522284113.

(18) Miller, S. M.; Wofsy, S. C.; Michalak, A. M.; Kort, E. A.; Andrews, A. E.; Biraud, S. C.; Dlugokencky, E. J.; Eluszkiewicz, J.; Fischer, M. L.; Janssens-Maenhout, G.; et al. Anthropogenic emissions of methane in the United States. *Proceedings of the National Academy of Sciences* **2013**. DOI: 10.1073/pnas.1314392110.

(19) Prather, M. J.; Hsu, J.; DeLuca, N. M.; Jackman, C. H.; Oman, L. D.; Douglass, A. R.; Fleming, E. L.; Strahan, S. E.; Steenrod, S. D.; Søvde, O. A.; et al. Measuring and modeling the lifetime of nitrous oxide including its variability. *Journal of Geophysical Research: Atmospheres* **2015**, *120* (11), 5693-5705. DOI: https://doi.org/10.1002/2015JD023267.

(20) Smith, C.; Nicholls, Z. R. J.; Armour, K.; Collins, W.; Forster, P.; Meinshausen, M.; Palmer, M. D.; Watanabe, M. *The Earth's Energy Budget, Climate Feedbacks, and Climate Sensitivity Supplementary Material. In Climate Change 2021: The Physical Science Basis. Contribution of Working Group I to the Sixth Assessment Report of the Intergovernmental Panel on Climate Change*; 2021. https://www.ipcc.ch/.

(21) Miller, J. B.; Tans, P. P. Calculating isotopic fractionation from atmospheric measurements at various scales. *Tellus B: Chemical and Physical Meteorology* **2003**, *55* (2), 207-214. DOI: 10.3402/tellusb.v55i2.16697.

(22) Sherwood, O. A.; Schwietzke, S.; Arling, V. A.; Etiope, G. Global Inventory of Gas Geochemistry Data from Fossil Fuel, Microbial and Burning Sources, version 2017. *Earth Syst. Sci. Data* **2017**, *9* (2), 639-656. DOI: 10.5194/essd-9-639-2017.

(23) Lan, X.; Basu, S.; Schwietzke, S.; Bruhwiler, L. M. P.; Dlugokencky, E. J.; Michel, S. E.; Sherwood, O. A.; Tans, P. P.; Thoning, K.; Etiope, G.; et al. Improved Constraints on Global Methane Emissions and Sinks Using δ13C-CH4. *Global Biogeochemical Cycles* **2021**, *35* (6), e2021GB007000. DOI: https://doi.org/10.1029/2021GB007000.

(24) Oh, Y.; Bruhwiler, L.; Lan, X.; Basu, S.; Schuldt, K.; Thoning, K.; Michel, S. E.; Clark, R.; Miller, J. B.; Andrews, A.; et al. *CarbonTracker CH4 2023*. NOAA Global Monitoring Laboratory, 2023. https://gml.noaa.gov/ccgg/carbontracker-ch4/carbontracker-ch4-2023 (accessed.

(25) Oh, Y.; Zhuang, Q.; Welp, L. R.; Liu, L.; Lan, X.; Basu, S.; Dlugokencky, E. J.; Bruhwiler, L.; Miller, J. B.; Michel, S. E.; et al. Improved global wetland carbon isotopic signatures support post-2006 microbial methane emission increase. *Communications Earth & Environment* **2022**, *3* (1), 159. DOI: 10.1038/s43247-022-00488-5.

(26) Saueressig, G.; Crowley, J. N.; Bergamaschi, P.; Brühl, C.; Brenninkmeijer, C. A. M.; Fischer, H. Carbon 13 and D kinetic isotope effects in the reactions of CH4 with O(1 D) and OH: New laboratory measurements and their implications for the isotopic composition of stratospheric methane. *Journal of Geophysical Research: Atmospheres* **2001**, *106* (D19), 23127-23138. DOI: https://doi.org/10.1029/2000JD000120.

(27) King, S. L.; Quay, P. D.; Lansdown, J. M. The 13C/12C kinetic isotope effect for soil oxidation of methane at ambient atmospheric concentrations. *Journal of Geophysical Research: Atmospheres* **1989**, *94* (D15), 18273-18277. DOI: https://doi.org/10.1029/JD094iD15p18273.

(28) Cantrell, C. A.; Shetter, R. E.; McDaniel, A. H.; Calvert, J. G.; Davidson, J. A.; Lowe, D. C.; Tyler, S. C.; Cicerone, R. J.; Greenberg, J. P. Carbon kinetic isotope effect in the oxidation of methane by the hydroxyl radical. *Journal of Geophysical Research: Atmospheres* **1990**, *95* (D13), 22455-22462. DOI: https://doi.org/10.1029/JD095iD13p22455.
(29) Saunois, M.; Stavert, A. R.; Poulter, B.; Bousquet, P.; Canadell, J. G.; Jackson, R. B.; Raymond, P. A.; Dlugokencky, E. J.; Houweling, S.; Patra, P. K.; et al. The Global Methane Budget 2000–2017. *Earth Syst. Sci. Data* **2020**, *12* (3), 1561-1623. DOI: 10.5194/essd-12-1561-2020.

Table S1. U.S. total annual net CH_4 emissions in Tg yr^{-1} derived from the current study with HYSPLIT-NAMS/GFS and WRF-STILT footprints. Uncertainties are 2σ errors. "n.a." indicates "not available".

Year	HYSPLIT-NAMS/GFS	WRF-STILT
2008	n.a.	51 ± 5
2009	n.a.	47 ± 5
2010	n.a.	46 ± 5
2011	n.a.	49 ± 5
2012	n.a.	48 ± 3
2013	n.a.	42 ± 4
2014	n.a.	46 ± 4
2015	53 ± 5	45 ± 4
2016	49 ± 4	47 ± 3
2017	52 ± 7	51 ± 5
2018	55 ± 6	n.a.
2019	59 ± 7	n.a.
2020	57 ± 7	n.a.
2021	56 ± 6	n.a.

Table S2. Comparison of derived U.S. methane emissions from this study versus previous studies. The listed uncertainties are either 2σ or the range reported by the referred study. "in situ" represent ground-based and aircraft observations, whereas GOSAT and TROPOMI represent CH_4 retrievals from those two satellites.

**Studies**	**Time periods**	**Models**	**Observations**	**Emissions ($Tg\ yr^{-1}$)**
This study	2015 - 2017	HYSPLIT-NAMS and WRF-STILT	in situ	50 ($\pm$ 6)
CT-CH4-2023	2015-2017	TM5	in situ	54
CAMS	2015-2017	TM5	in situ	36
Miller et al. (2013)	2007 - 2008	WRF-STILT	in situ	47
Global Carbon Project-CH4	2008 - 2017	multiple	in situ	43 ($\pm$ 13)
Turner et al. (2015)	2009 - 2011	GEOS-Chem	GOSAT	52.4 (50 - 53.8)
Maasakkers et al. (2021)	2010 - 2015	GEOS-Chem	GOSAT	42.4 (37 - 42.9)
Worden et al. (2022)	2019	GEOS-Chem	GOSAT	44.9 ($\pm$ 10.8)
Nesser et al. (2024)	2019	GEOS-Chem	TROPOMI	39.3 (38.2 - 40.3)
Global Carbon Project-CH4	2008 - 2017	multiple	GOSAT	38 ($\pm$ 17)
Lu et al. (2022)	2010 - 2017	GEOS-Chem	GOSAT+insitu	46.3 (40.2 - 48.4)

Table S3. Multi-year average monthly CH_4 emissions from the U.S. and U.S. regions between 2015 and 2017. Emissions are in Tg yr^{-1} with 2σ uncertainties. Region definitions were shown in Fig. S10.

Month	US	NE	SE	CN	CS	M	W
1	57 ± 13	8.3 ± 2.8	3.8 ± 1.9	16 ± 4	24 ± 8	3.1 ± 2.0	2.3 ± 1.1
2	52 ± 7	7.4 ± 2.6	3.9 ± 2.0	15 ± 3	20 ± 5	3.0 ± 2.4	2.2 ± 1.1
3	48 ± 7	6.5 ± 1.8	4.7 ± 1.7	13 ± 3	19 ± 5	2.9 ± 2.6	1.8 ± 1.0
4	47 ± 8	5.4 ± 1.8	5.9 ± 1.7	12 ± 3	18 ± 4	3.3 ± 2.6	2.4 ± 1.1
5	43 ± 6	5.1 ± 1.4	4.3 ± 1.4	12 ± 2	16 ± 4	3.2 ± 2.4	2.2 ± 1.2
6	47 ± 4	4.7 ± 1.8	5.6 ± 1.5	15 ± 3	17 ± 2	3.4 ± 2.0	1.6 ± 0.9
7	51 ± 6	5.7 ± 1.9	5.2 ± 2.8	18 ± 4	17 ± 3	3.3 ± 2.5	1.8 ± 1.4
8	52 ± 5	6.0 ± 1.7	5.2 ± 2.3	18 ± 3	17 ± 3	3.8 ± 3.1	2.5 ± 1.6
9	50 ± 5	6.1 ± 1.7	6.8 ± 3.0	16 ± 2	14 ± 2	4.7 ± 3.3	2.5 ± 1.2
10	49 ± 11	5.6 ± 1.6	5.9 ± 4.1	15 ± 3	17 ± 5	3.3 ± 3.2	1.5 ± 0.9
11	50 ± 10	5.5 ± 1.9	5.6 ± 2.5	15 ± 3	19 ± 6	3.3 ± 2.5	1.3 ± 1.1
12	50 ± 10	7.2 ± 2.2	6.0 ± 1.8	14 ± 5	18 ± 4	2.8 ± 2.2	1.9 ± 1.1

Table S4. The Bayesian Information Criterion scores for different combinations of geospatial datasets.

Spatial datasets	BIC Scores	ΔBIC (relative to the lowest BIC score)
Oil production	74466	162
Natural gas production	74533	229
Population	74732	428
Oil production and natural gas production	74489	185
Natural gas production and population	74489	185
Oil production, natural gas production, and population	74304	0

Table S5. Estimated U.S. source signatures and their weights in the calculation of U.S. average isotopic signatures of $^{13}CH_4$ for fossil fuel and microbial sources.

Sector	Subsector	Source signature	Weight	
			option 1	option 2
Fossil fuel	Coal	-52.2	0.25	
	Oil and gas	-41.5	0.70	
	Other industrial activities	-44.1	0.05	
Microbial sources[a]	Wetland	-66.9	0.53	0.32
	Ruminants	-66.7	0.28	0.40
	Rice	-62.2	0.02	0.03
	Waste	-56.3	0.15	0.22
	Termites	-65.1	0.01	0.02
	Wild animals	-66.5	0.01	0.01
biomass burning		-24.9		

Notes:

a. Two sets of flux weights were considered for calculating U.S. average microbial source signatures. Option 1 represents flux weights when using TEM wetland CH_4 emissions, whereas Option 2 represents weights when using Kaplan wetland CH_4 emissions.

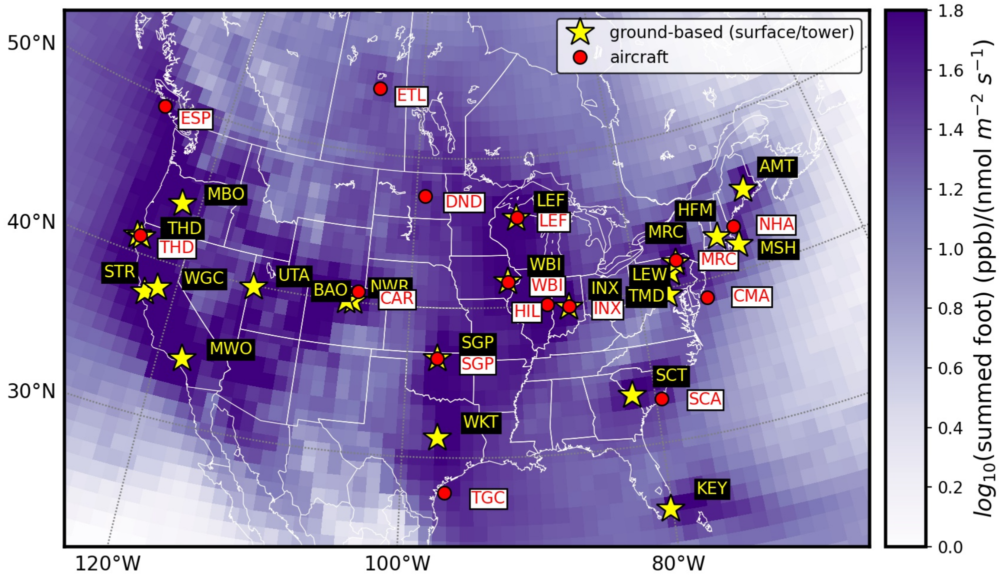

Fig. S1. Site map for atmospheric CH_4 observations used in inverse modeling of this study. Yellow stars represent ground-based measurements, whereas red circles represent aircraft measurements. The purple shading represents the summed 10-day sensitivity (footprints) of measurements made in 2015 - 2017 on a logarithm scale.

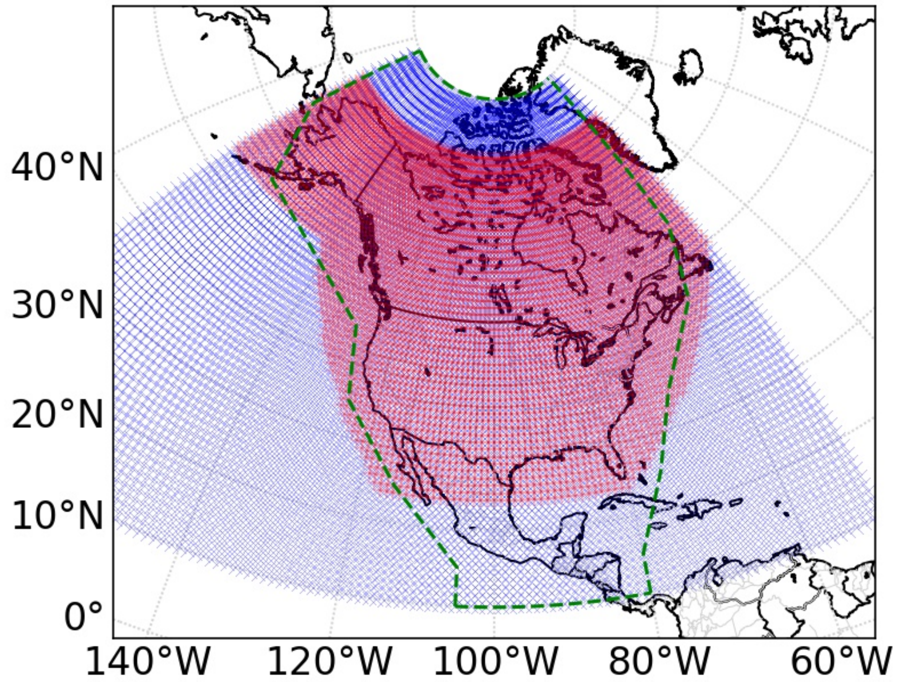

Fig. S2. A map indicating our model domain. Grid cells with blue pluses indicate the domain of our footprints. Red crosses denote grid cells to be optimized in the inversion. The green dashed line denotes the horizontal boundaries for identifying particles entering or exiting the inversion model domain.

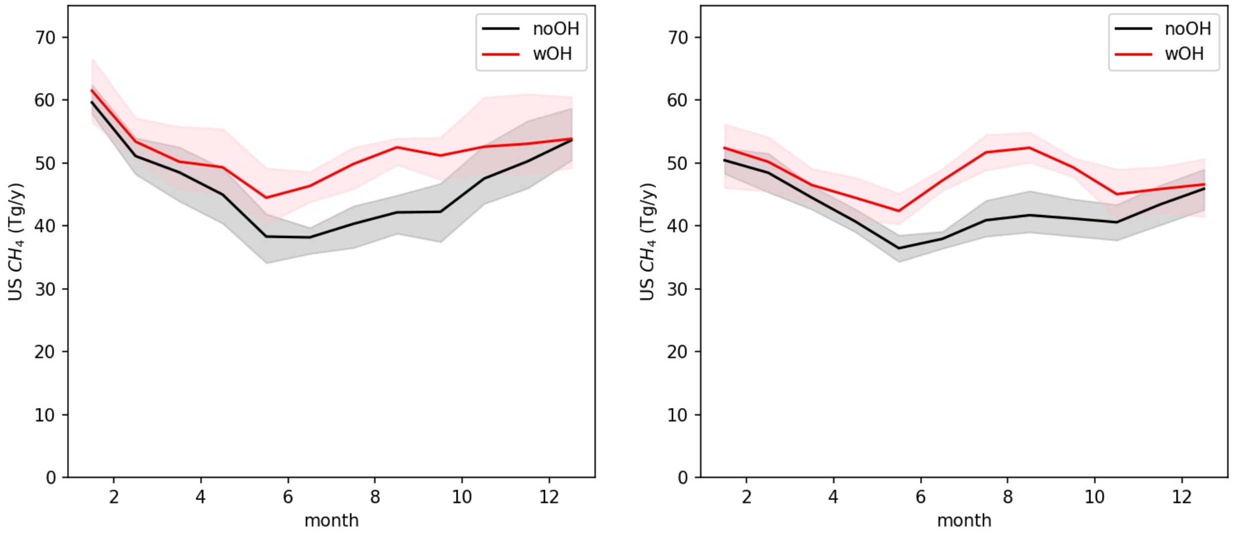

Fig. S3. Multi-year average seasonal cycle of U.S. CH_4 emissions between 2015 and 2017 with (red) and without (black) consideration of OH losses in the HYSPLIT-NAMS/GFS model (left panel) and WRF-STILT model (right panel). The colored shadings indicate the spread of the six scenarios with three different backgrounds and two different prior emissions.

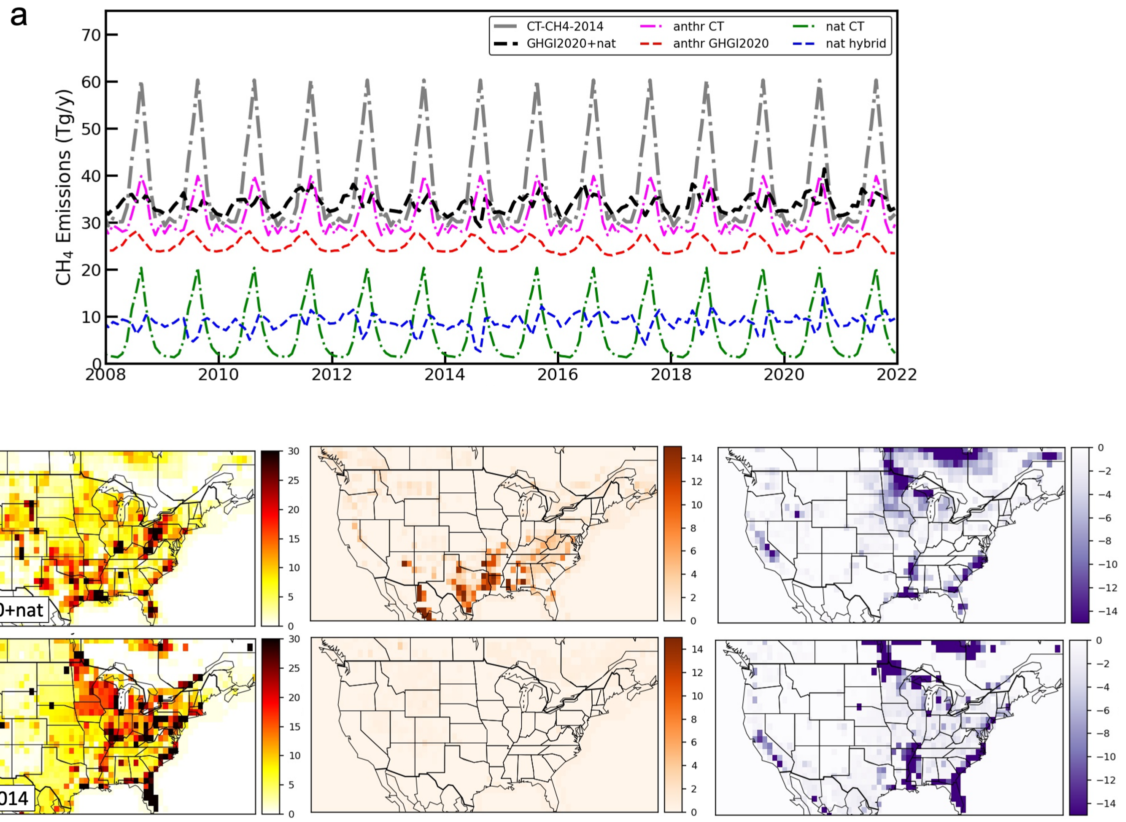

Fig. S4. Two prior CH_4 emissions considered in this study: CT-CH4-2014 (dash dotted lines) and GHG2020+nat (dashed lines). (a) Monthly U.S. total, anthropogenic, and natural emissions of CH_4 from both priors. Total, anthropogenic, and natural emissions from CT-CH4-2014 are denoted as gray, magenta, and green dash dotted lines, whereas those from GHG2020+nat are denoted as black, red, and blue dashed lines. (b) Prior emissions maps (in nmol m^{-2} s^{-1}) for multi-year average annual emissions between 2015 and 2017 (left panels) and their winter-summer differences: positive difference are shown in middle panels and negative differences are shown in right panels.

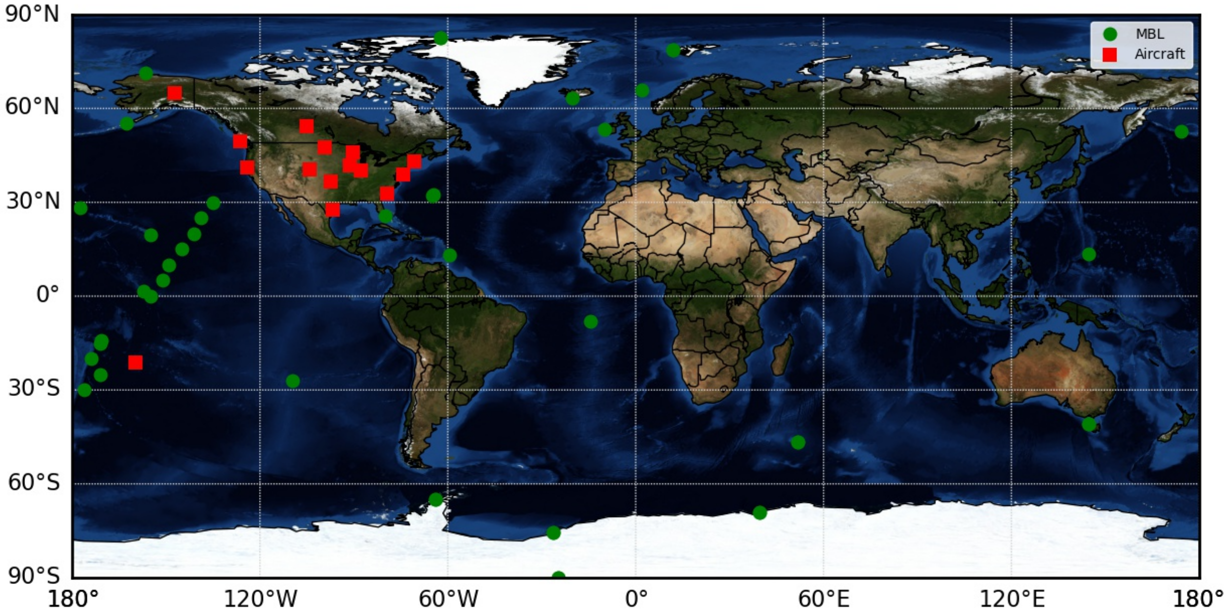

Fig. S5. Sites used to construct the 4D empirical background. Green symbols indicate marine boundary layer sites, whereas red symbols indicate aircraft sites.

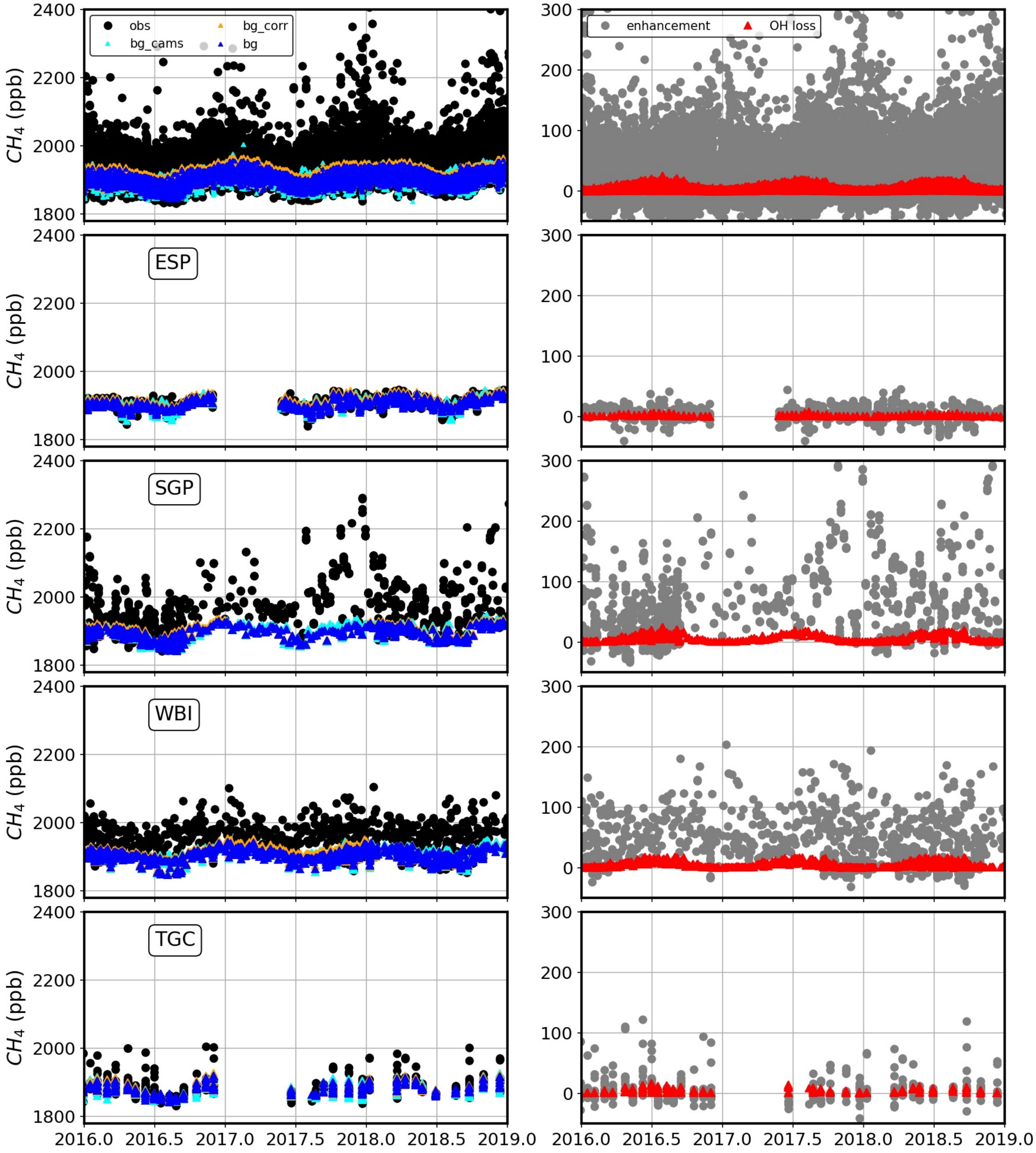

Fig. S6. (Left Panels) Observed and estimated background CH_4 mole fractions. Black circles are observations, whereas cyan, pink, and blue symbols are estimated backgrounds without OH corrections. (Right Panels) Estimated enhancements by subtracting background from observations (gray) and estimated OH loss in background (red). The top panels are all sites, whereas the lower panels are some selected sites.

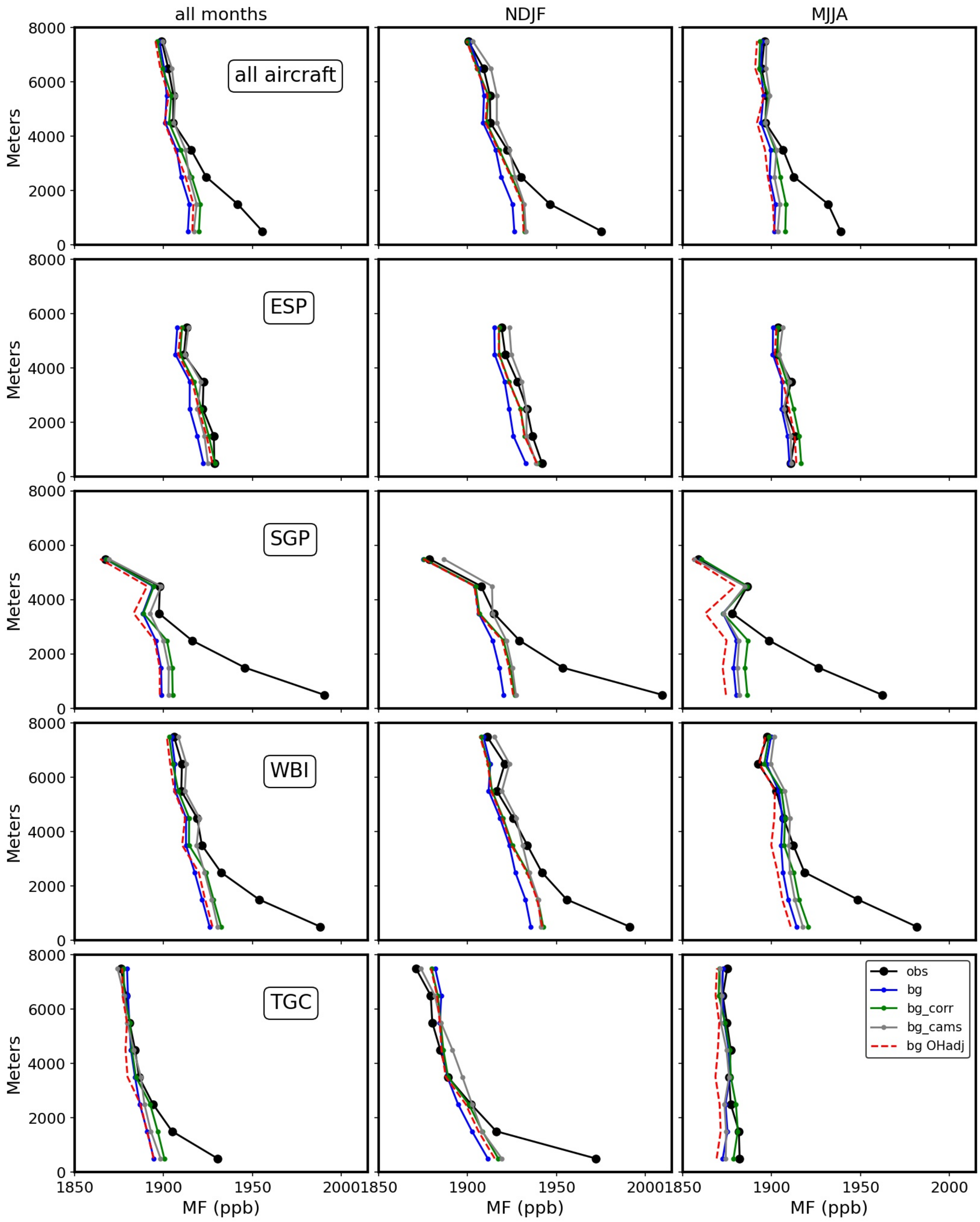

Fig. S7. Vertical gradients in aircraft observations and their estimated backgrounds, average for all months (left panels), for Nov – Feb (middle panels), and for May – Aug (right panels).

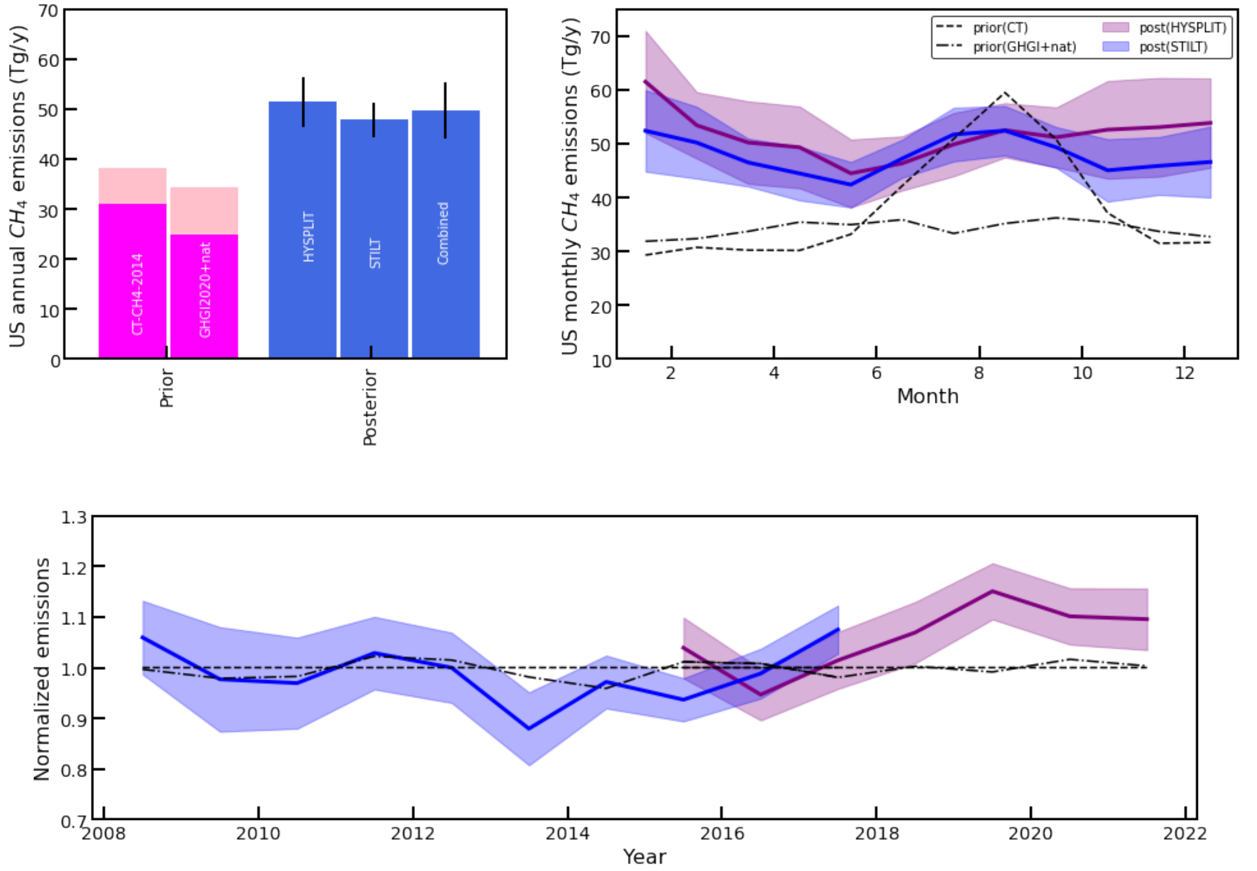

Fig. S8. Comparison between our posterior and prior U.S. CH_4 total net emissions. (Upper left) Multi-year average U.S. annual total net CH_4 emissions between 2015 and 2017: prior estimates in magenta and pink (magenta for anthropogenic and pink for natural emissions) and posterior estimates in blue with errorbars indicating 2σ uncertainties. (Upper right) Multi-year average U.S. monthly CH_4 emissions from our prior and posterior estimates between 2015 and 2017. Colored shadings represent 2σ errors. The black dashed line represents the prior CT-CH4-2014, whereas the black dash dotted line indicates the prior GHG2020+nat. (Lower panel) Normalized U.S. annual CH_4 emissions from prior and posterior estimates. The representations of lines and shadings are consistent with those in the upper right panel.

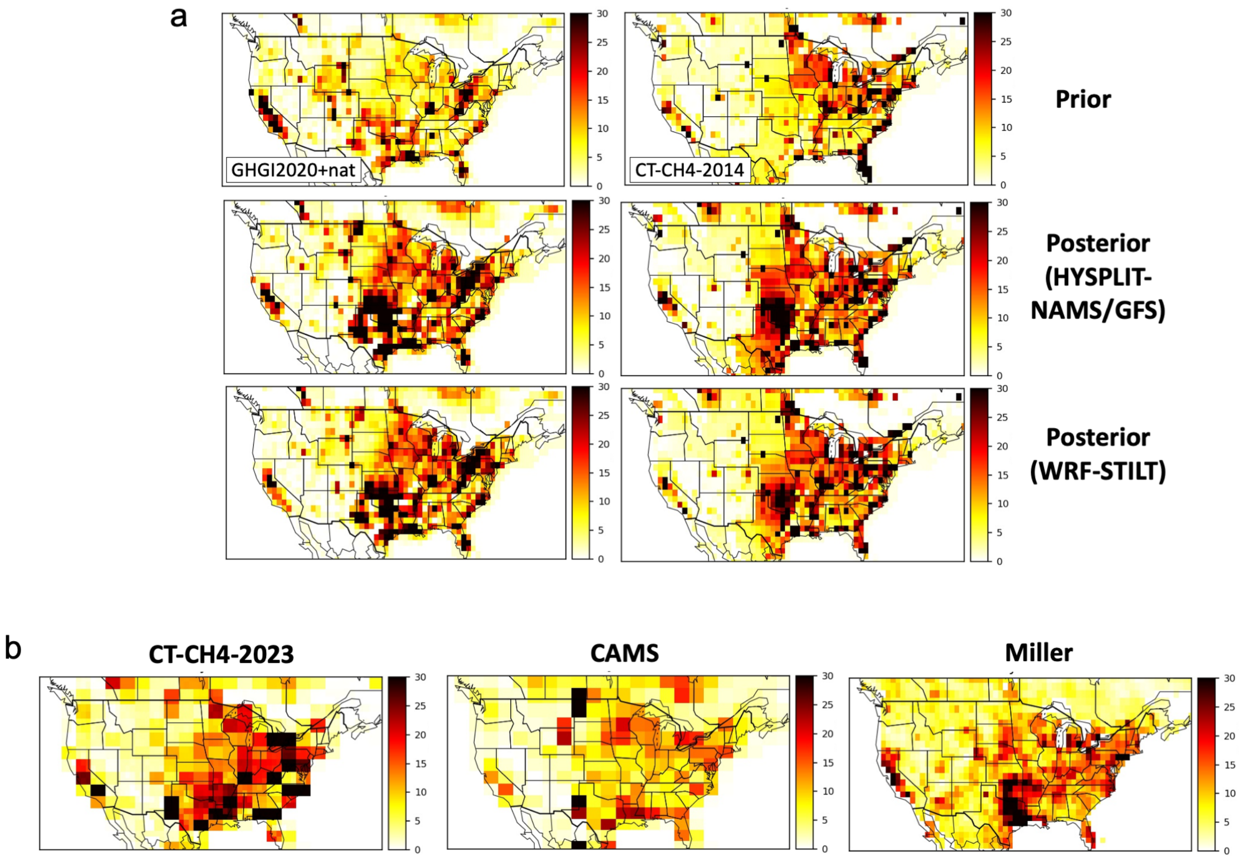

Fig. S9. Grid-scale multi-year annual average emissions of CH_4 in nmol m^{-2} s^{-1}. (a) Prior and posterior estimates from this study for 2015 - 2017. (b) Posterior estimates from CT-CH4-2023 and CAMS for 2015-2017, and Miller et al. (2013) for 2007-2008.

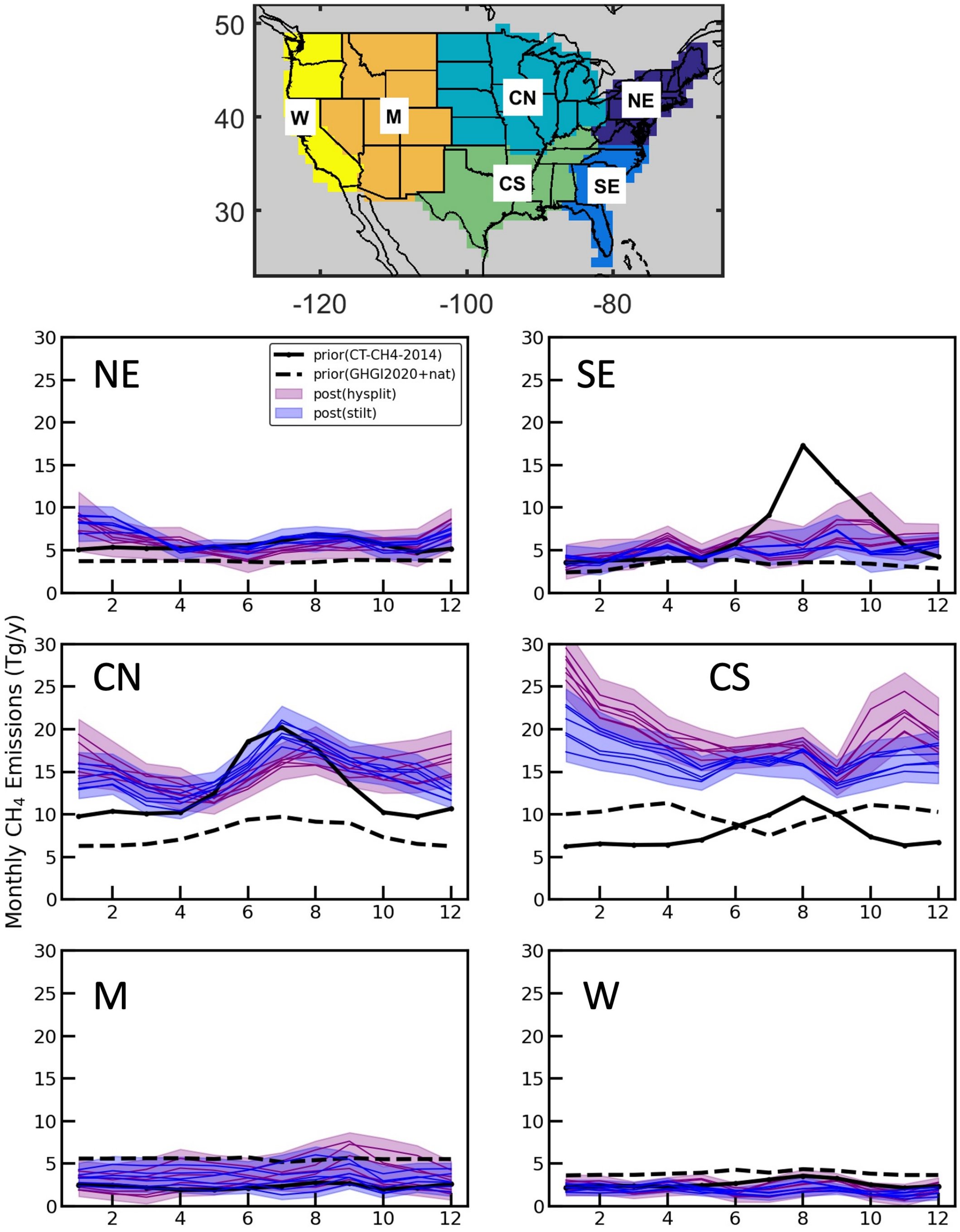

Fig. S10. Multi-year average monthly CH_4 emissions from U.S. regions between 2015 – 2017. Regions are defined in the top panel. In the lower six panels, blue lines and shadings represent posterior runs driven by the HYSPLIT-NAMS/GFS footprints, whereas purple lines and shadings represent posterior estimates using the WRF-STILT footprints. Blue and Purple shadings denote 2σ uncertainties. Black solid lines denote multi-year average monthly emissions from the prior CT-CH4-2014, whereas black dashed lines denote multi-year average monthly emissions from the prior GHGI2020+nat.

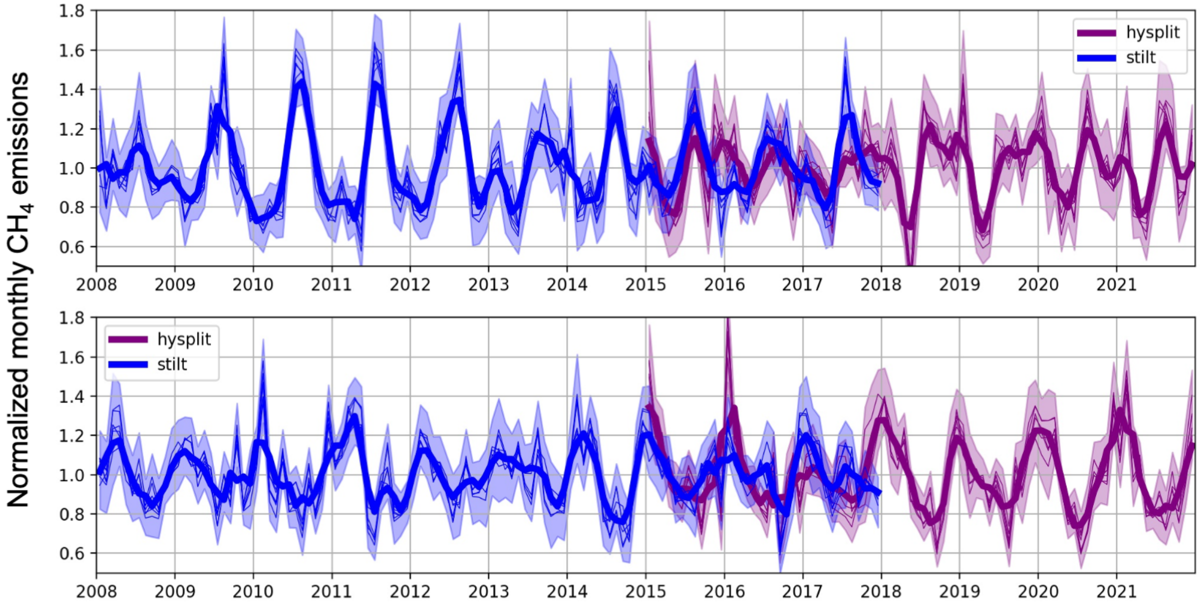

Fig. S11. Normalized monthly CH_4 emissions for CN (top panel) and CS (lower panel) regions. Thin lines indicate ensemble means, whereas thick lines indicate their 3-monthly running means. Color shadings indicate 2σ uncertainties.

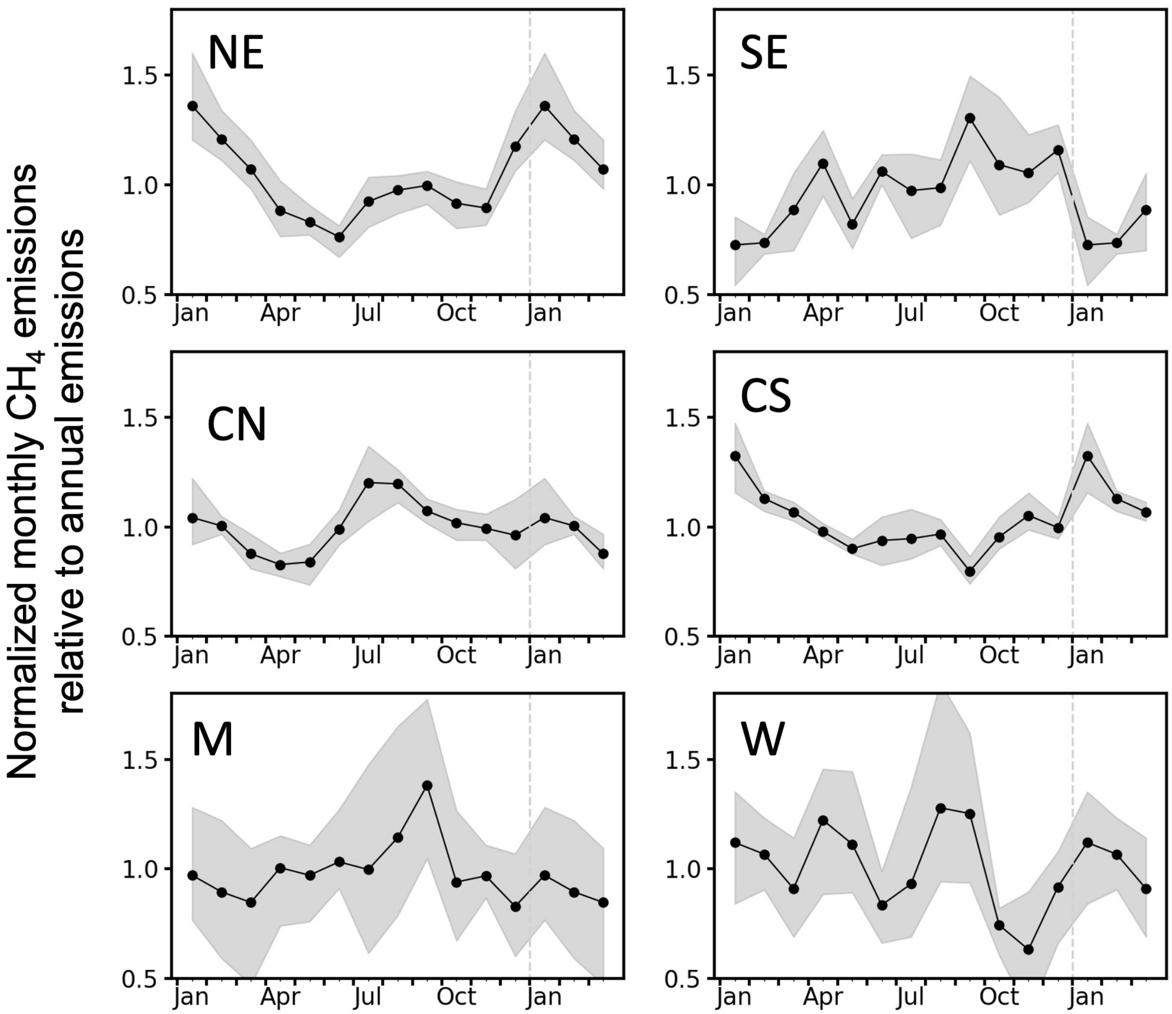

Fig. S12. Normalized monthly CH_4 emissions relative to their annual emissions for the six regions defined in Fig. S10 and averaged between 2015 – 2017. Jan – Mar emissions were repeated at the end to improve the overall visualization.

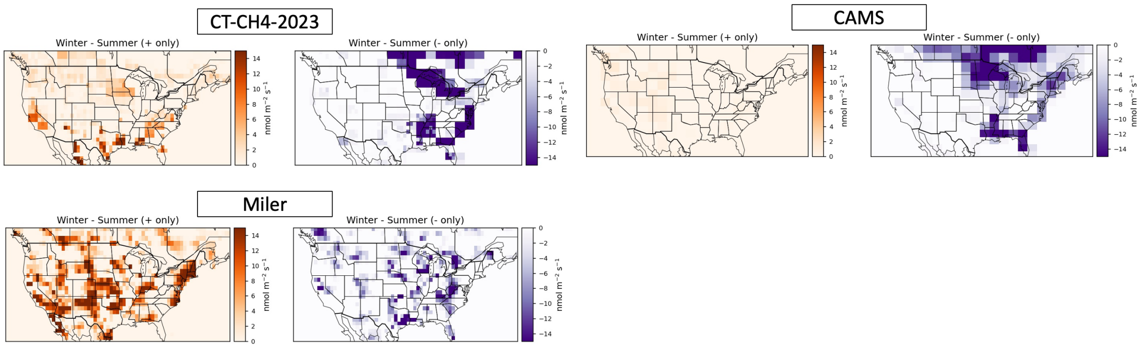

Fig. S13. Winter-summer differences in CH_4 emissions derived from CT-CH4-2023, CAMS, and Miller et al. (2013). Maps with warm (orange) colors indicate regions with enhanced winter emissions, whereas maps with cold (purple) colors indicate regions with enhanced summer emissions.

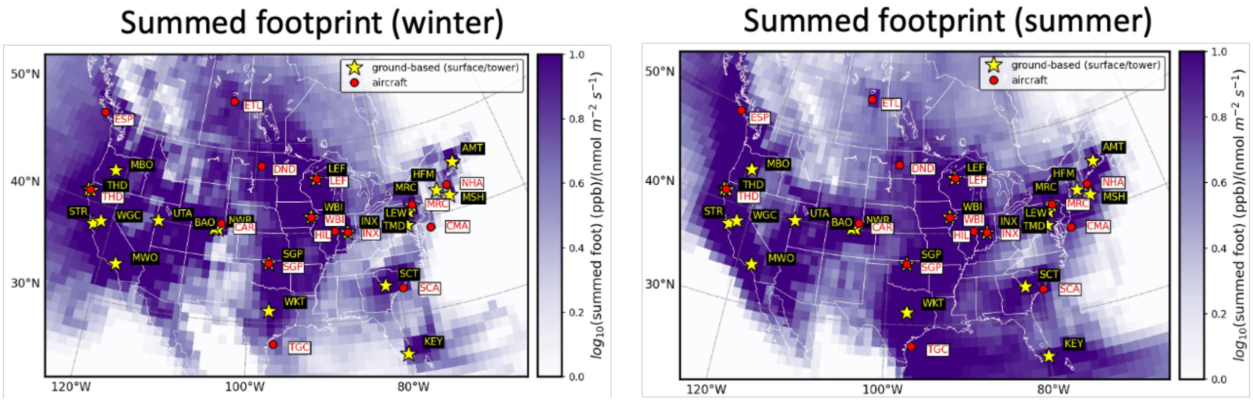

Fig. S14. Winter and summer footprints summed from observations included in our inverse analysis between 2015 – 2017. Yellow stars represent ground-based measurements, whereas red circles represent aircraft measurements.

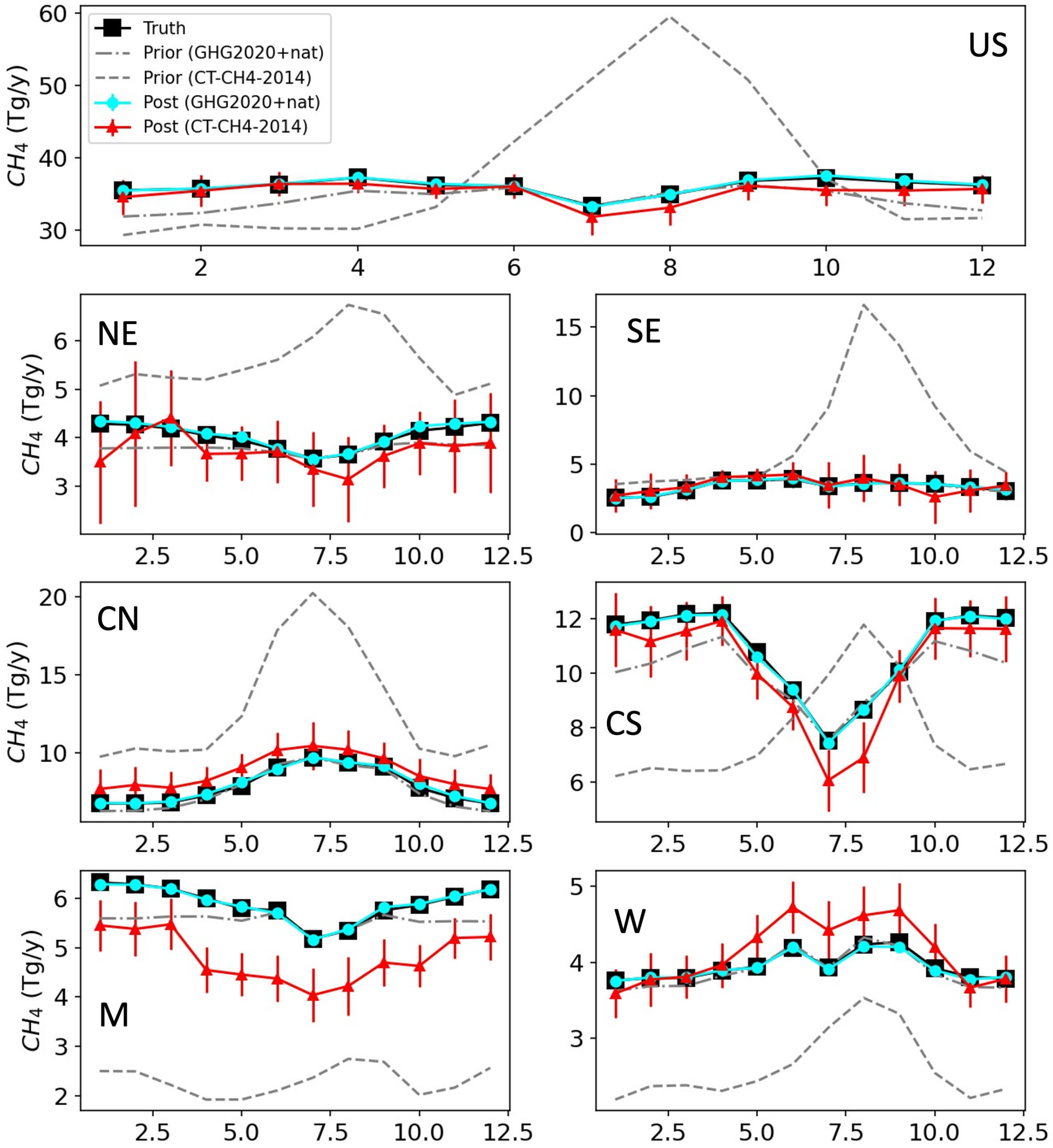

Fig. S15. Derived national and regional multi-year average monthly CH_4 emissions from OSSEs between 2015 – 2017. Black squares connected with black lines represent the true emissions. Gray dash and dash dotted lines represent the two prior emissions. Blue circles connected with blue lines and red triangles connected with red lines represent the derived posterior emissions. Errorbars indicate 2σ errors.

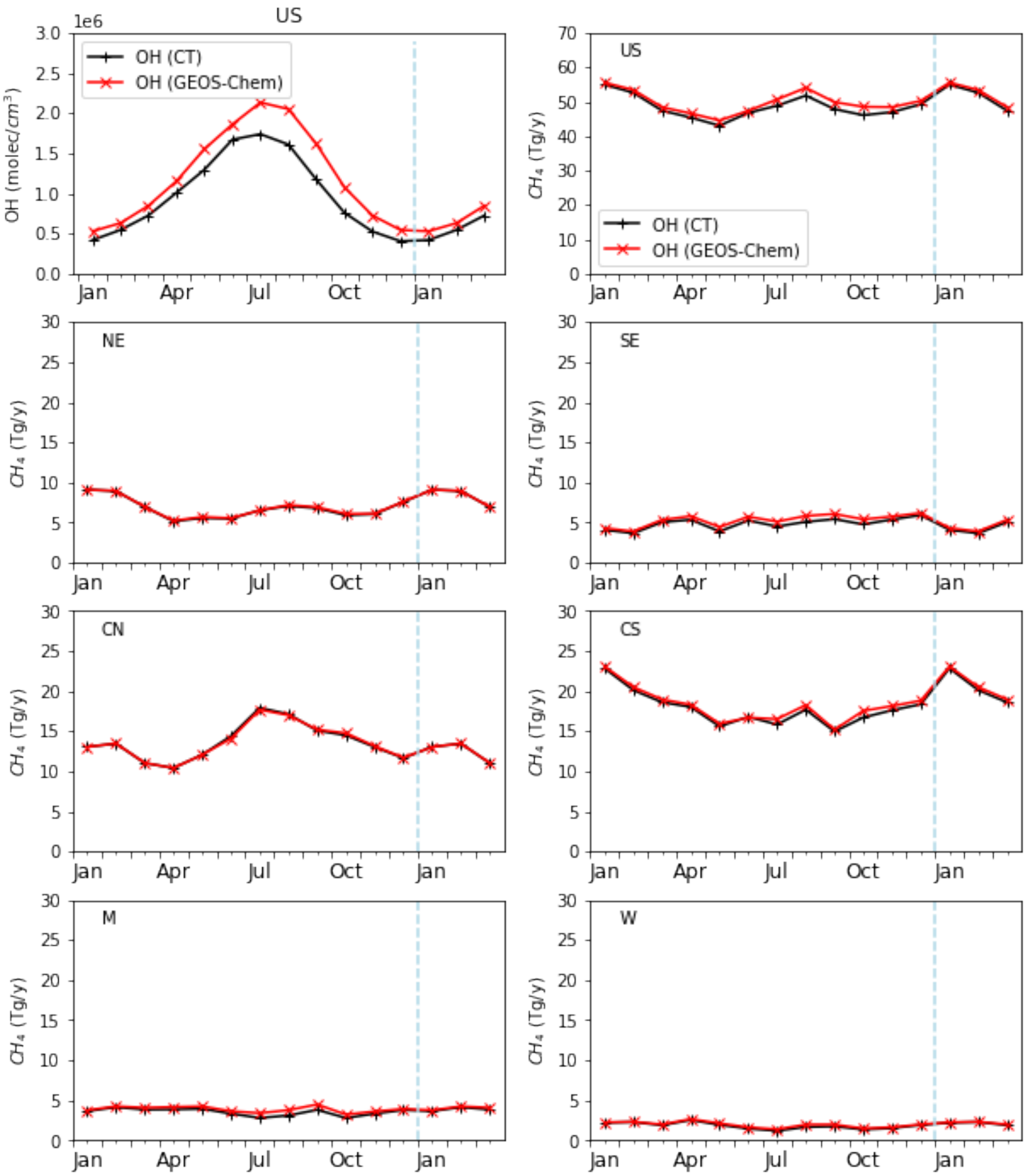

Fig. S16. Sensitivity test of inversely derived CH_4 emissions to different OH fields. (Top Left Panel) Median OH concentrations in the atmosphere above the U.S. from CT-CH4-2023 and GEOS-Chem models. (Other panels) U.S. national and regional monthly CH_4 emissions averaged between 2015 – 2017, derived from inversions using the two OH products and the same model setup (WRF-STILT footprints, background "bg_corr", and the prior GHGI2020+nat).

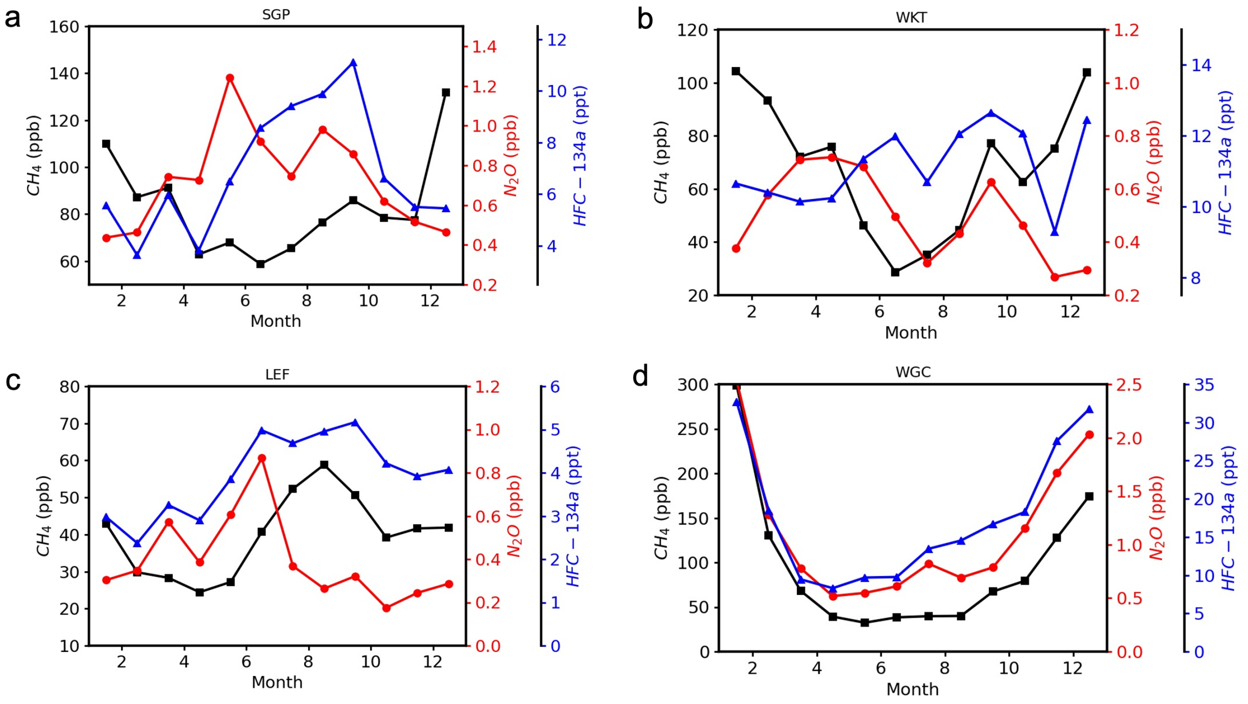

Fig. S17. Observed monthly atmospheric mole fraction enhancements of CH_4 (black), N_2O (red), and HFC-134a (blue) averaged between 2008 and 2021 at Southern Great Plains, Oklahoma (SGP), Moody, Texas (WKT), Park Falls, Wisconsin (LEF), and Walnut Grove, California (WGC). Site locations are indicated in Fig. S1.

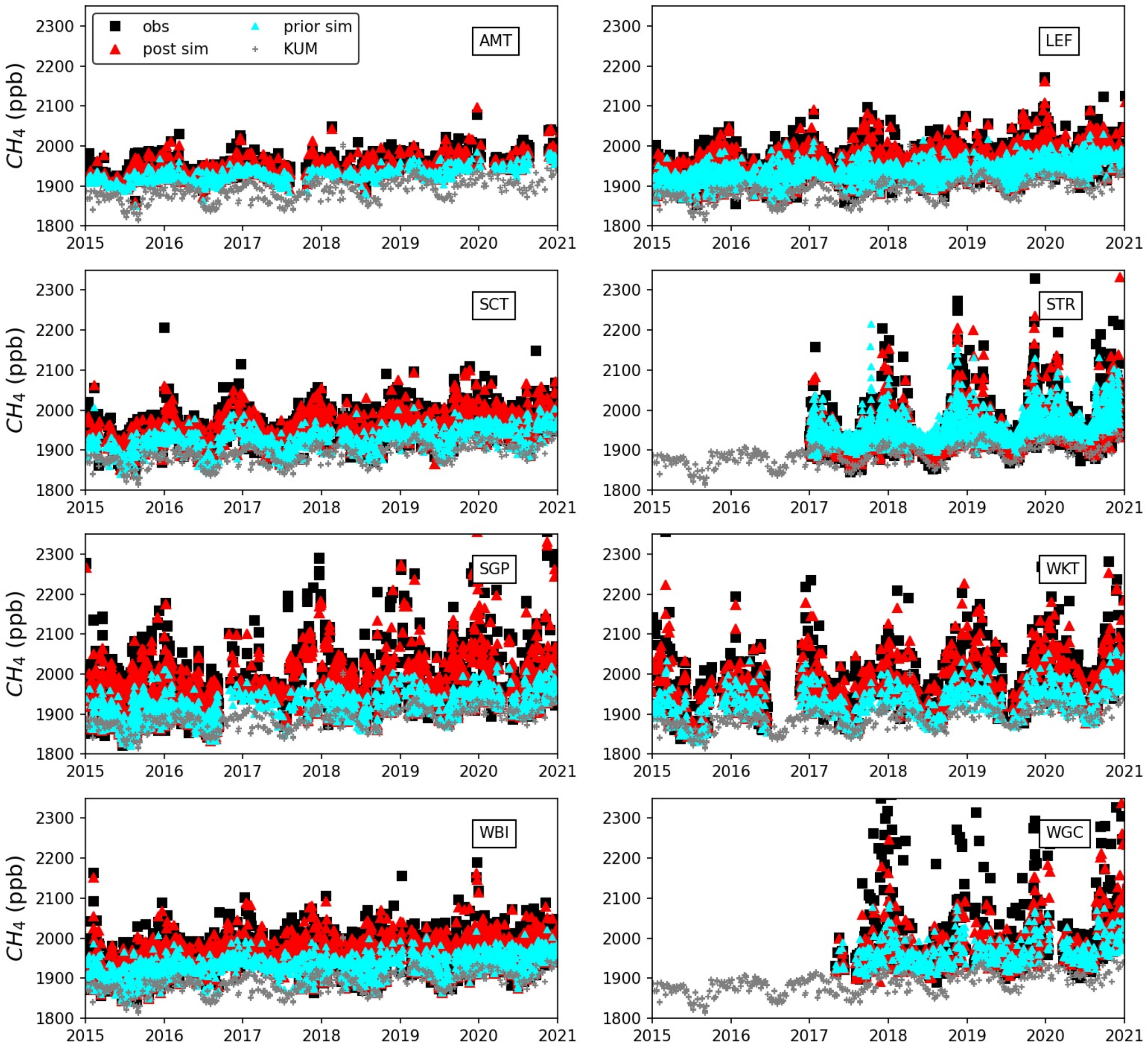

Fig. S18. Observed (black squares) and simulated (cyan and red triangles) mole fractions at selected tower sites across the U.S. Locations of selected sites (denoted by site codes) are shown in Fig. S1. Simulated mole fractions shown here are computed from prior GHGI2020+nat (cyan) and its corresponding posterior emissions (red), convolved with HYSPLIT-NAMS/GFS footprints. Observed mole fractions from a marine boundary layer site at KUM, Hawaii (in gray) were also shown.

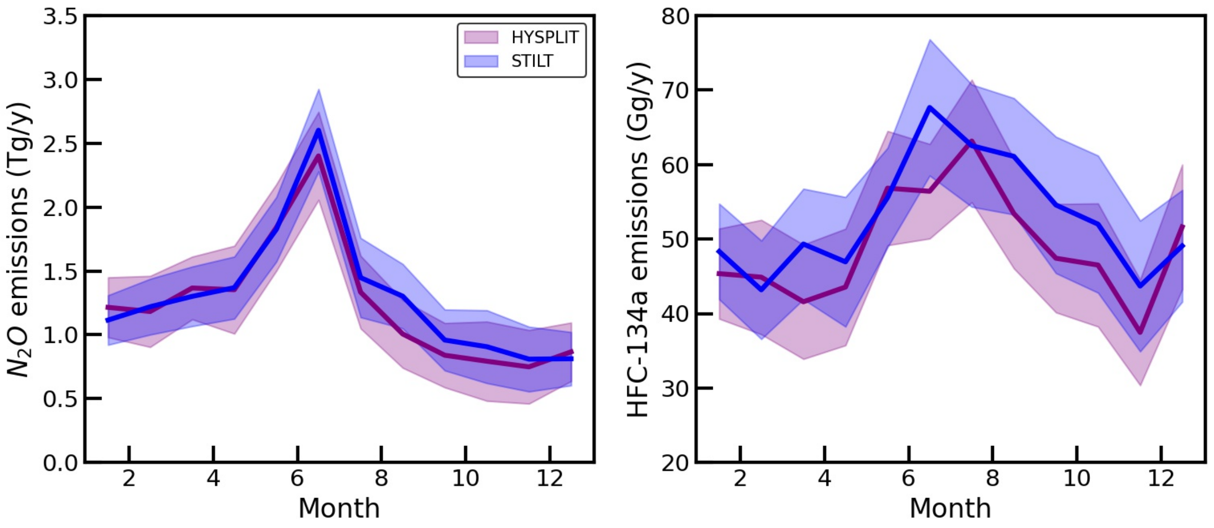

Fig. S19. Multi-year monthly average U.S. emissions of N_2O and HFC-134a derived from our inverse models for 2015 - 2017.

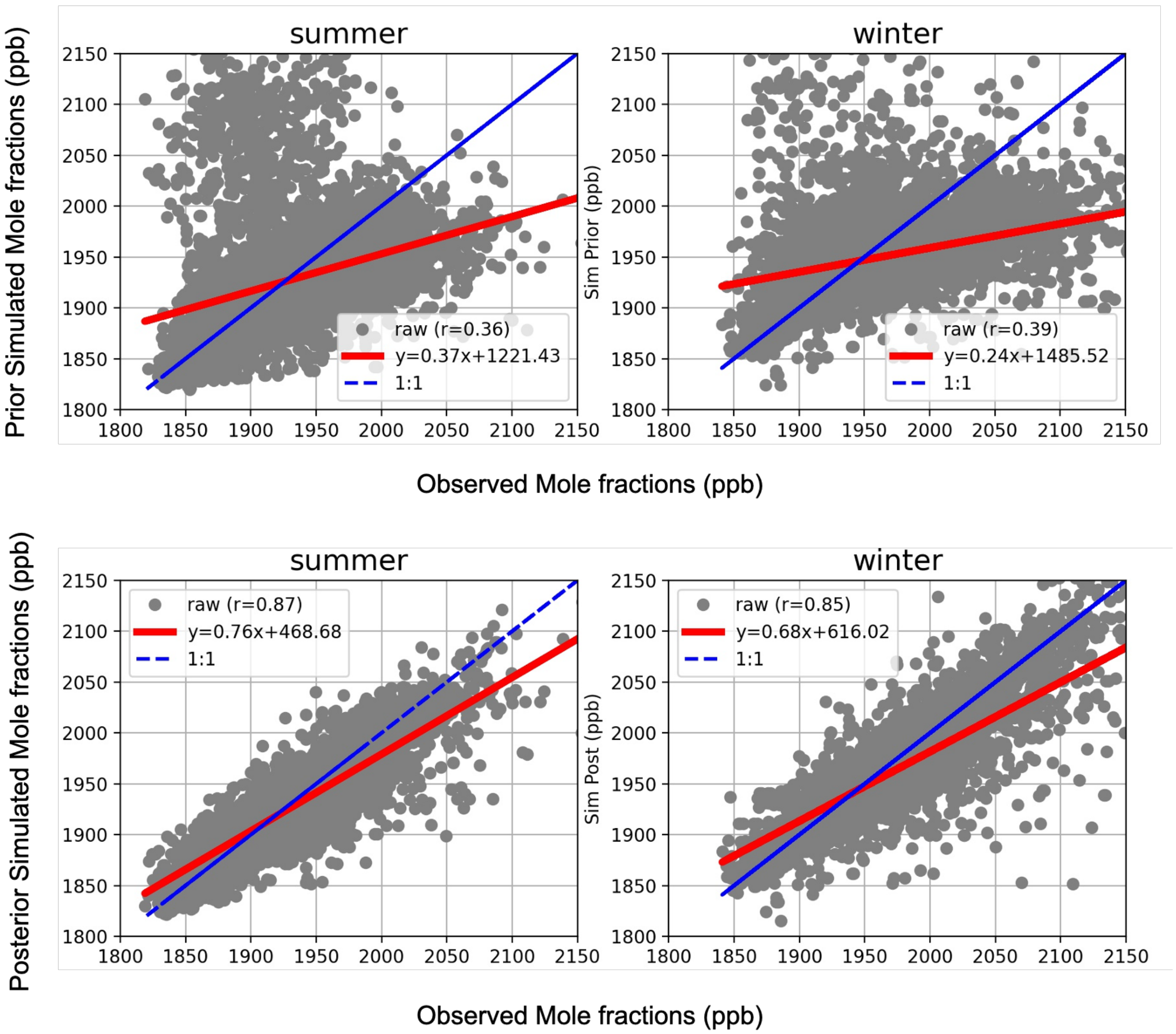

Fig. S20. Simulated versus observed mole fractions of CH_4 for winter and summer using data used in inversions. The upper panels are simulations with prior emissions and the lower panels are simulations with posterior emissions. Blue lines indicate 1:1 lines. Red lines are linear regressions. Simulated mole fractions shown here were computed from prior GHGI2020+nat and its corresponding posterior emissions, convolved with HYSPLIT-NAMS/GFS footprints.

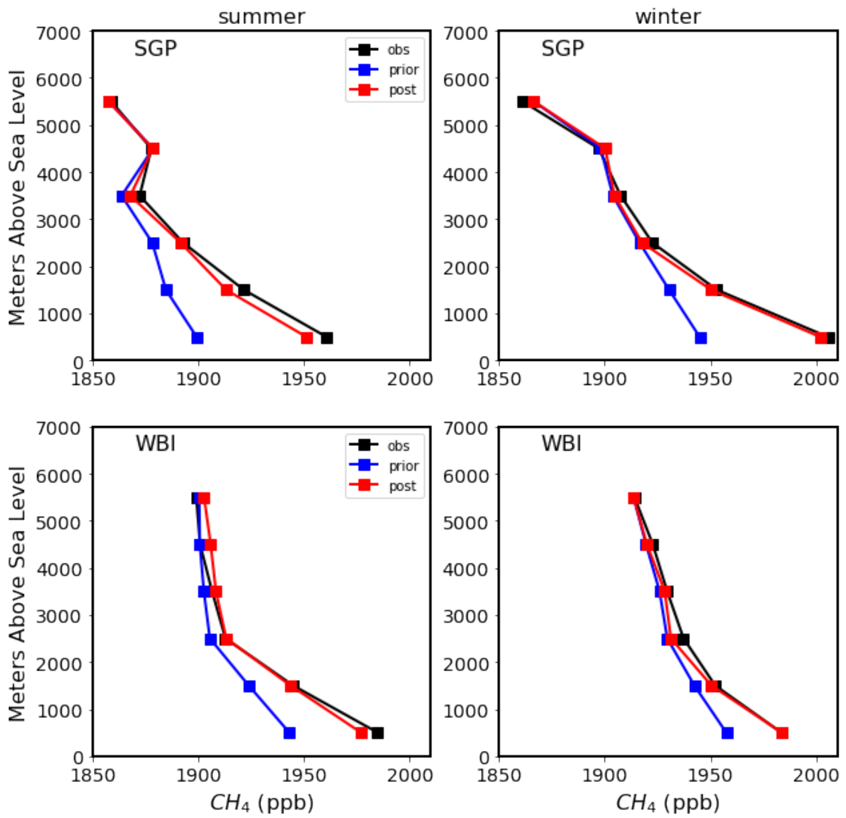

Fig S21. Average vertical profiles in observed CH_4 mole fractions and simulated CH_4 mole fractions using prior and posterior emissions for summer and winter at SGP and WBI aircraft sites between 2015 – 2021.

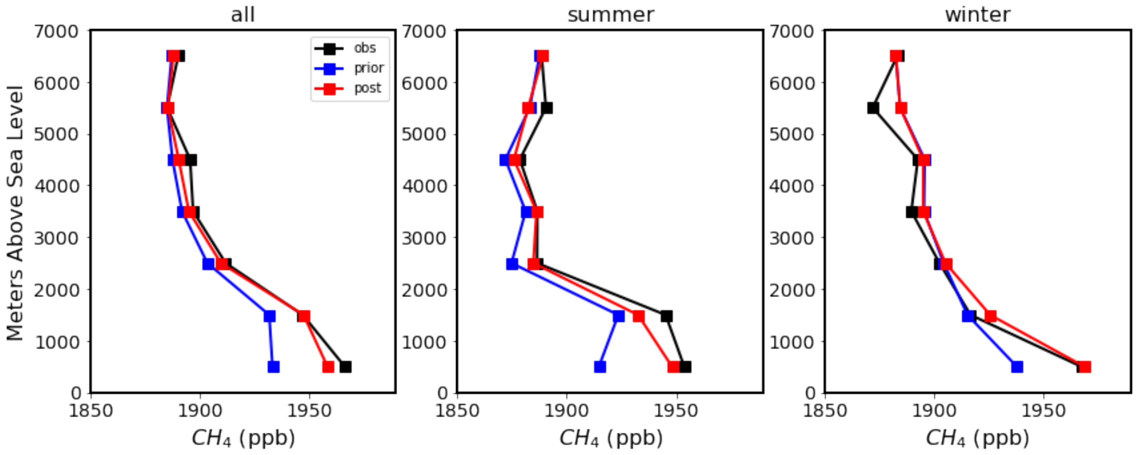

Fig S22. Average vertical profiles in observed CH_4 mole fractions and simulated CH_4 mole fractions using prior and posterior emissions for summer and winter with independent aircraft data, including ACT-America and east coast campaigns data.

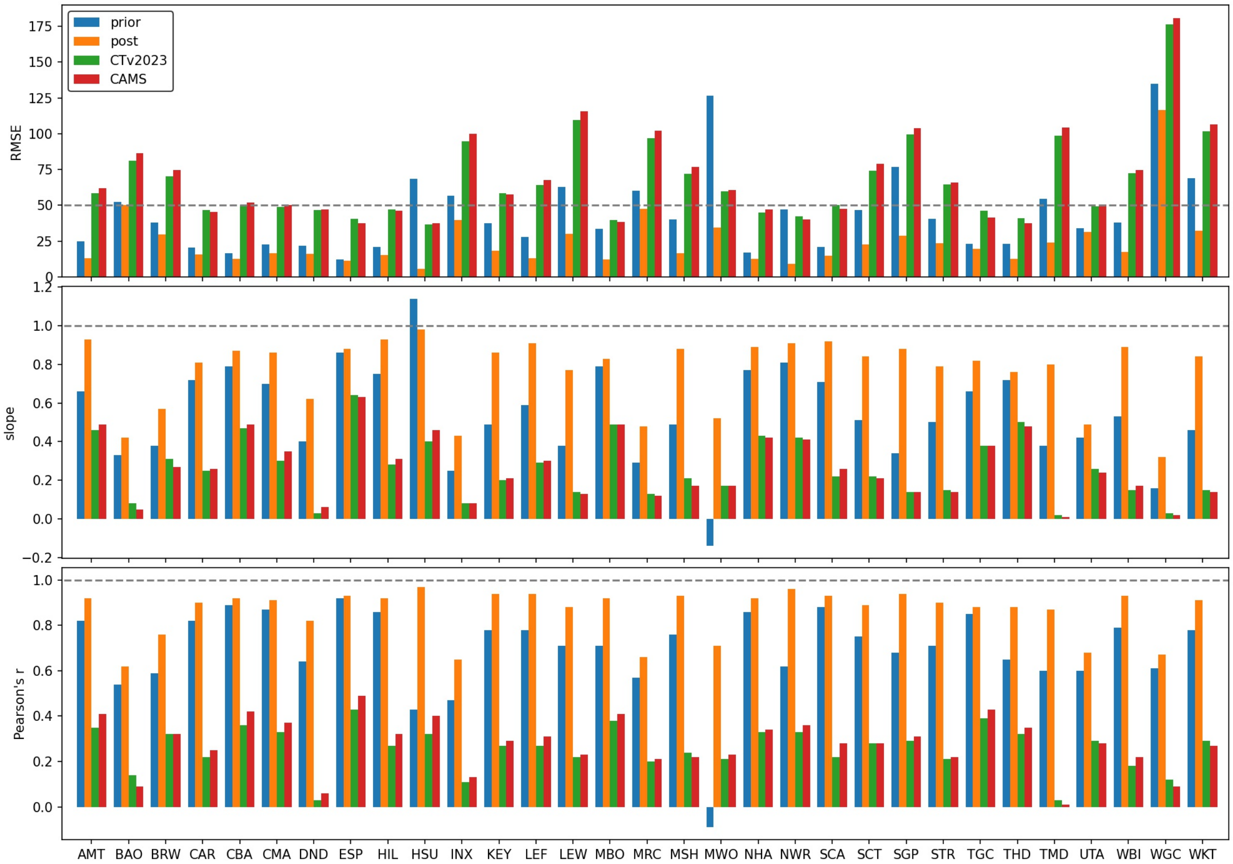

Fig. S23. Summary statistics for atmospheric CH_4 observations used in our inversions. Root mean square errors in ppb (top panel), linear regression slopes (middle panel), and Pearson's correlation coefficients between simulated mole fractions and observations. Blue and orange bars represent the statistics for the prior and posterior simulations from this study, whereas green and red bars represent statistics for posterior simulations from CT-CH4-2023 and CAMS. Locations of individual sites are indicated in Fig. S1.

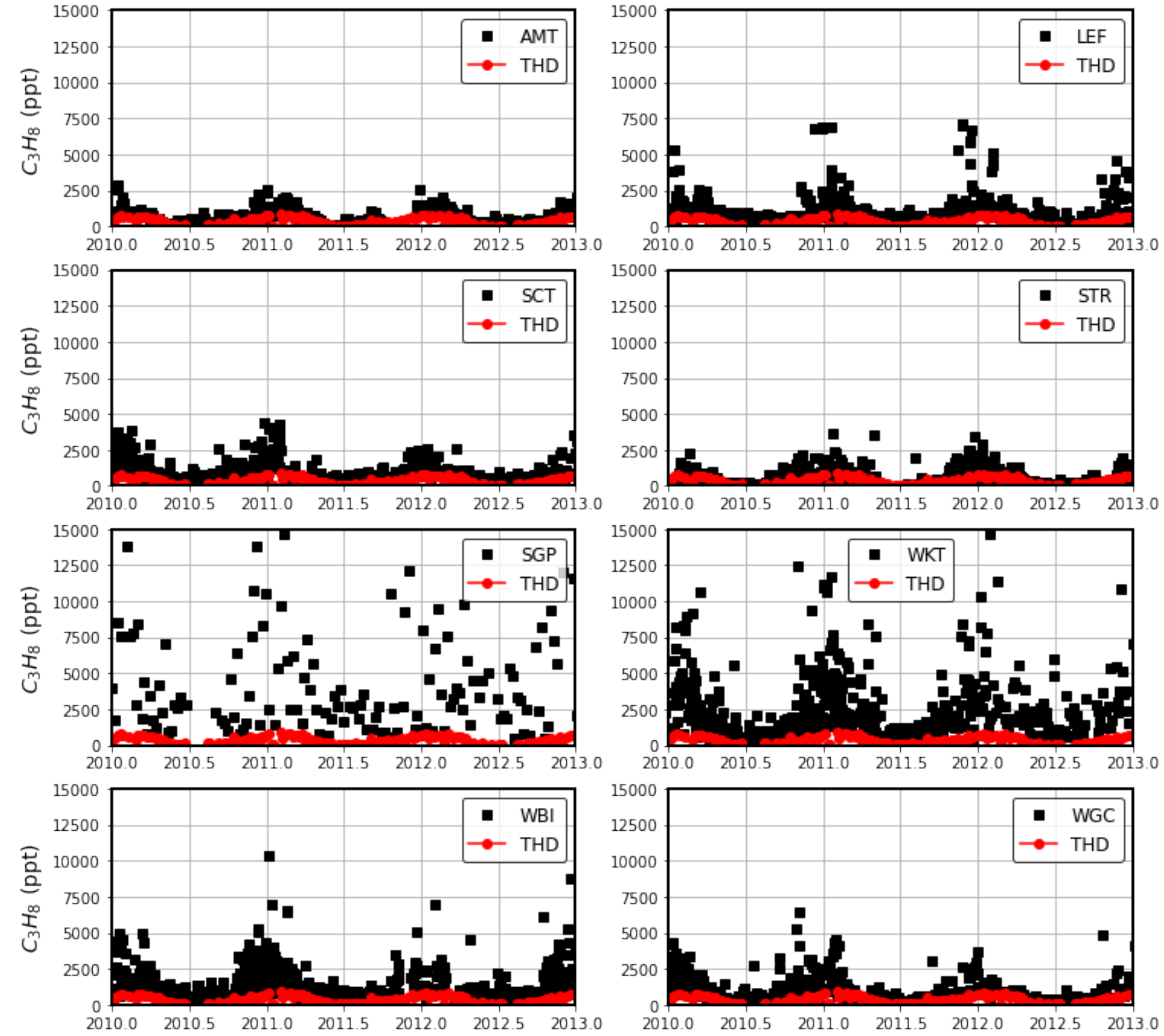

Fig. S24. Time series of observed C_3H_8 mole fractions at selected tower sites over the U.S. (black symbols) in comparison with C_3H_8 mole fractions at an upwind background site at Trinidad Head (THD), California. Site locations are indicated in Fig. S1.

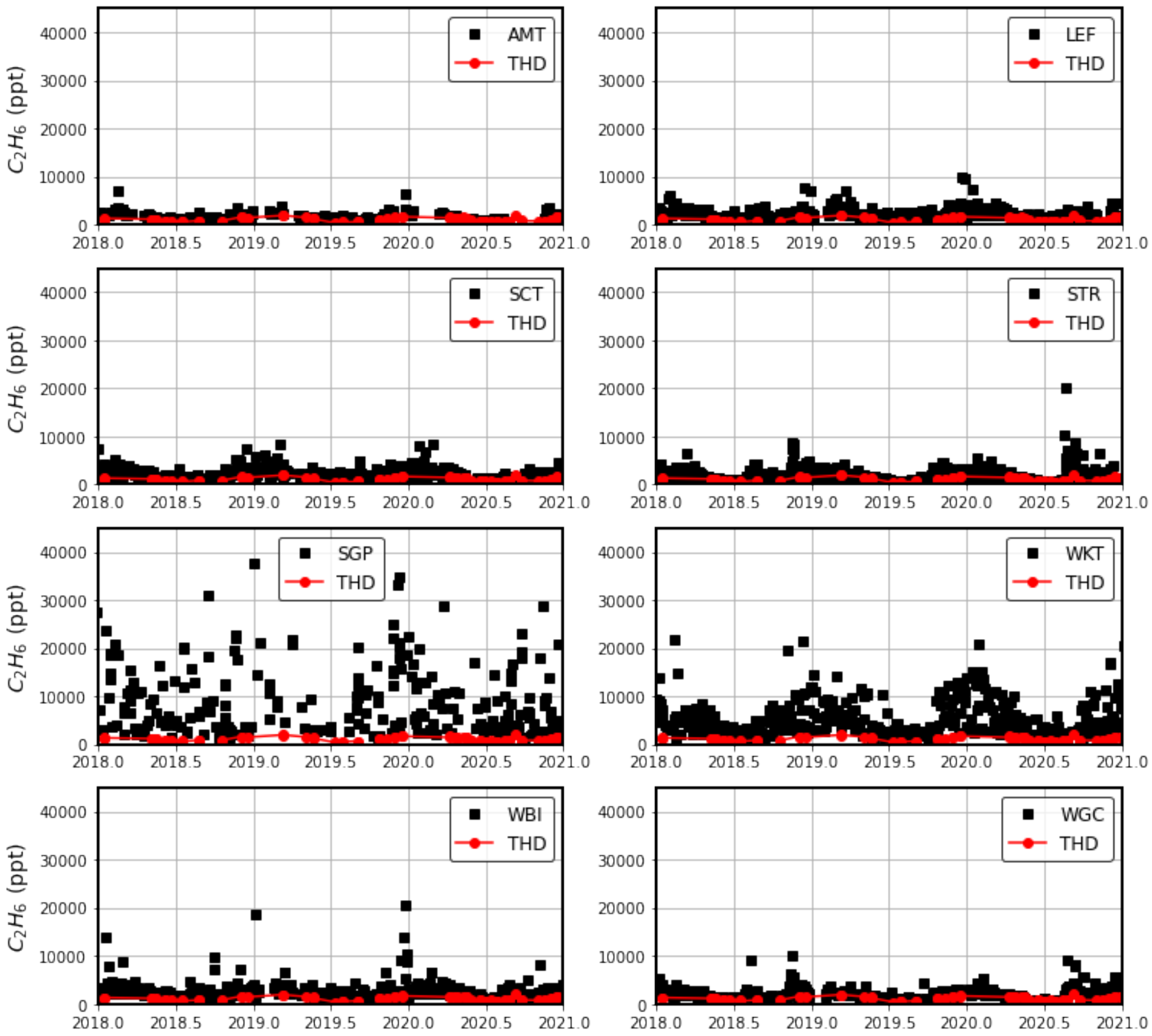

Fig. S25. Time series of observed C_2H_6 mole fractions at selected tower sites over the U.S. (black symbols) in comparison with C_3H_8 mole fractions at an upwind background site at Trinidad Head (THD), California. Site locations are indicated in Fig. S1.

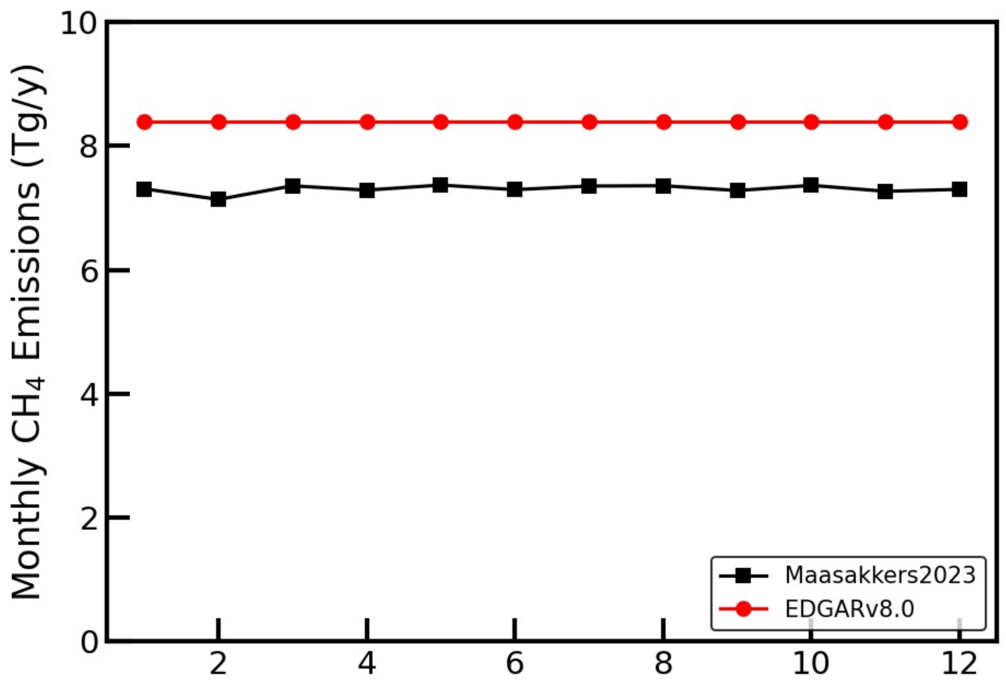

Fig. S26. Multi-year monthly average U.S. CH_4 emissions from the oil and gas sector represented in inventories between 2012 and 2017. The black symbols represent the gridded inventory from Maasakkers et al (2023), which is consistent with the US EPA's GHGI submitted to the United Nations in 2020. The red symbols are estimates from the Emissions Database for Global Atmospheric Research version 8.0 (EDGARv8.0).

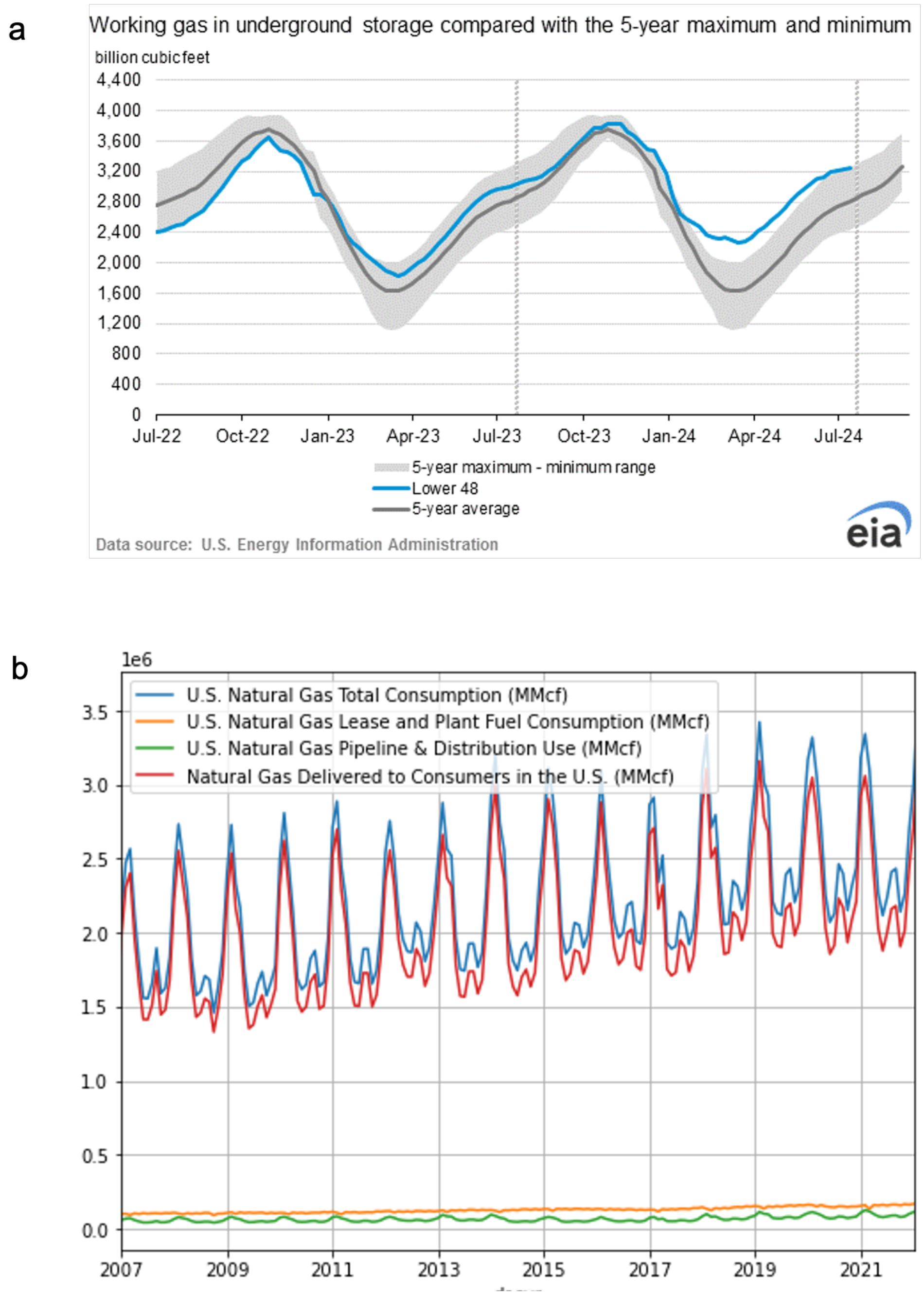

Fig. S27. U.S. natural gas underground storage (a) and consumption (b). Panel a is a figure from the U.S. Energy Information Administration (EIA) weekly natural gas storage report for week ending July 2026, 2024 (Date Access: August 8, 2024). Panel b shows monthly natural gas consumption in million cubic feet based on data published in the U.S. EIA.

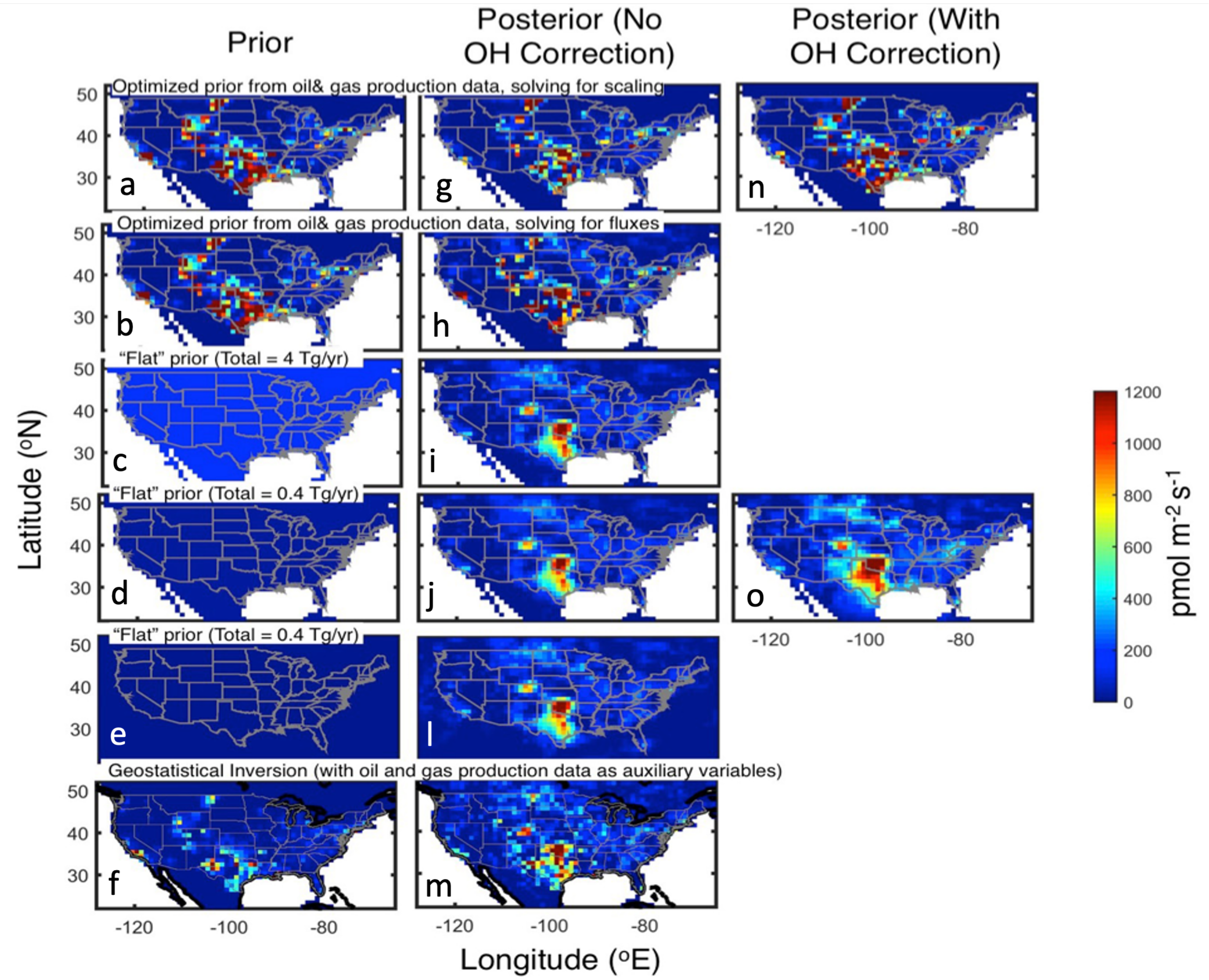

Fig. S28. U.S. emissions of C_3H_8 derived from Bayesian inversions with different priors (a-e) and a Geostatistical inversion (f) with and without considerations in the OH losses. In the Bayesian inversion framework, the first, third, and fourth rows show results with optimization on scaling factors of fluxes, whereas the second and fifth rows display posterior results with optimization on fluxes.

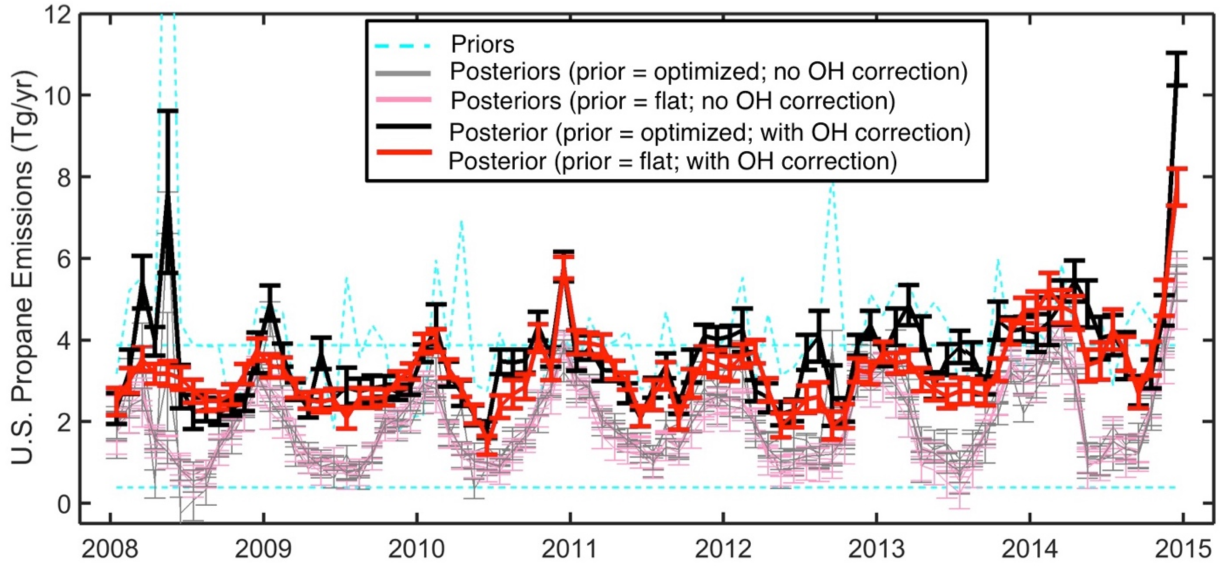

Fig. S29. Derived monthly U.S. total C_3H_8 emissions using Bayesian inversions with different prior emissions and with and without considerations in the OH losses. The cyan dashed lines indicate the considered prior emissions; gray and pink lines indicate posterior estimates without considerations of OH losses, whereas black and red lines denote posterior estimates when considering OH losses. Errorbars indicate 1σ errors.

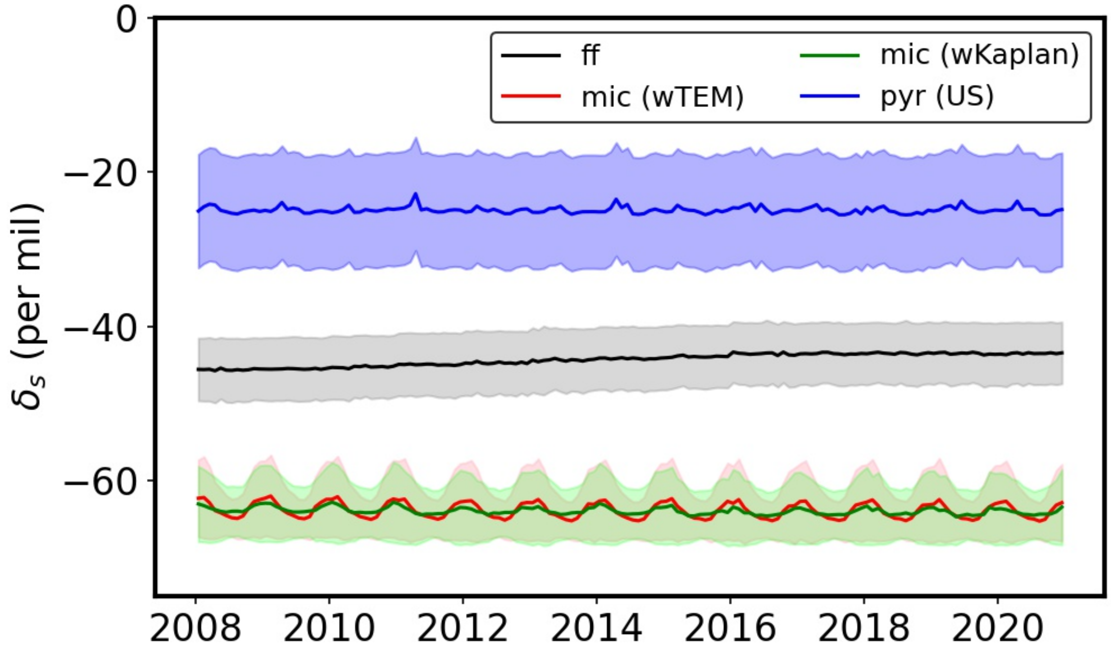

Fig. S30. Flux-weighted U.S. source signatures of fossil fuel (ff), microbial (mic), and pyrogenic (pyr) sources with their 1σ uncertainties. The microbial source signatures were estimated with considerations of two different wetland CH_4 emissions.

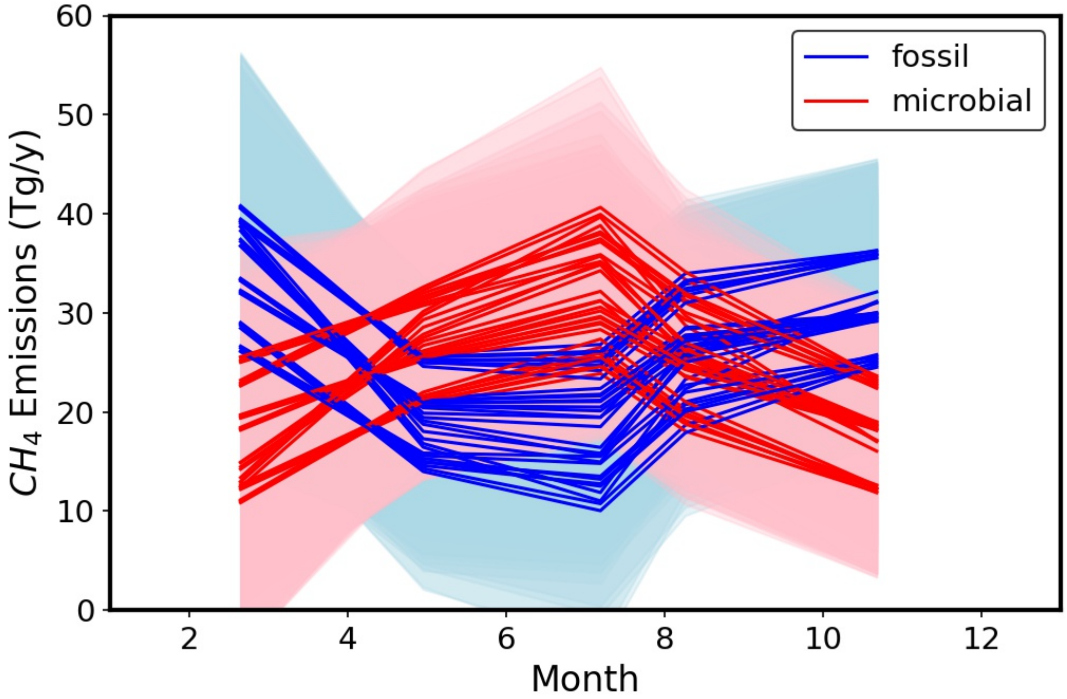

Fig. 31. Estimated U.S. fossil fuel and microbial CH_4 emissions. Solid lines indicate the best estimates, whereas color shadings indicate their 1σ error.
